# Trade-off between canonical and unusual recombination sites promotes diversity and stability of gene cassette arrays of mobile integrons

**DOI:** 10.1038/s41598-026-36353-0

**Published:** 2026-01-24

**Authors:** Adrián Gonzales Machuca, María Carolina Molina, Verónica Elizabeth Álvarez, Eduardo José Carpio Díaz, María Paula Quiroga, Daniela Centrón

**Affiliations:** Laboratorio de Investigaciones en Mecanismos de Resistencia a Antibióticos, Facultad de Medicina, Instituto de Investigaciones en Microbiología y Parasitología Médica (IMPaM), Universidad de Buenos Aires (UBA), Consejo Nacional de Investigaciones Científicas y Técnicas (CONICET), Buenos Aires, Argentina

**Keywords:** Integrons, IntI1, Class 1 integrase, Unusual gene cassettes, attI1, Recombination, Genetics, Microbiology, Molecular biology

## Abstract

Mobile integrons are the most efficient mechanism of Gram-negative bacteria to resist antimicrobial changing pressures in the nosocomial niche. Integron’s integrases mediate site-specific recombination of distinct DNA structures such as *attI*, *attC* and *attG* sites. Here, we identified novel Δ*attI* sites as part of 26 unusual Δ*attI*-type gene cassettes conferring resistance to four different antibiotic families. Since scarce data are found related to their functionality, we investigated site-specific recombination mediated by IntI1 of *attI1*-*aadB*-*attI1*, *attI1*-*aadB*-Δ*attI1*_− 11_, *attC*_*aadA1*_-*aadB*-Δ*attI2*_− 238_, and *attC*_*aadA1*_-*ybeA*-Δ*attI2*_− 11_ genetic architectures. All proved to be functional with some displaying excision and insertion rates equal to canonical gene cassettes. Interestingly, gene cassettes with the same recombination site upstream and downstream of the ORF, i.e. either two equal *attI1* or two *attC* sites, had an excision frequency of more than 97%, which outlines a scenario in which the canonical gene cassette, once inserted, is almost instantaneously excised. These findings evidence that a trade-off between different recombination sites, including *attI*,* attC* and Δ*attI* sites in canonical and unusual Δ*attI*-type gene cassettes, is necessary to maintain a stable and diverse gene cassette array within the variable region of mobile integrons.

**Supplementary Information:**

The online version contains supplementary material available at 10.1038/s41598-026-36353-0.

## Introduction

**Fig. 1:**
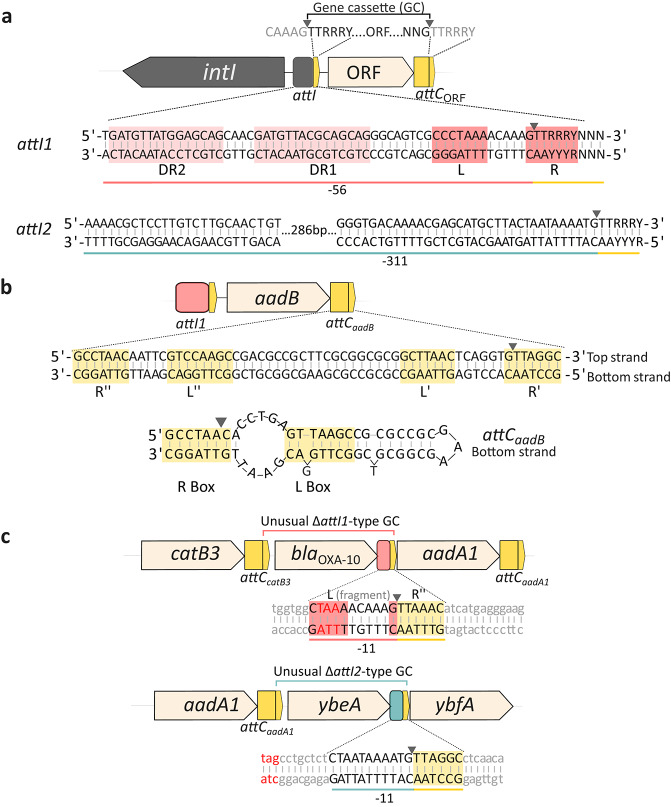
Integron and gene cassette structure. **(a)** Schematic representation of the structure of integrons, comprising the integrase gene (*intI*), and the primary recombination site (*attI*). The general structure of a gene cassette is schematically represented over the ORF gene cassette. The nucleotide sequences for *attI1* and *attI2* are shown, highlighting the *attI1* Direct Repeats (DR1 and DR2) and the L and R boxes. Specific structural elements have not yet been defined for *attI2.* Some studies have considered the *attI2* region to span approximately 311 bp, extending from the translation initiation codon of the *intI2* gene to the recombination crossover point. Grey arrowheads indicate the conserved “G” recombination crossover point **(b)** Structure of the *attC*_*aadB*_ site. Double stranded structure of the gene cassette (GC) *aadB* (DQ393784). The linear sequence of the *attC*_*aadB*_ site displays the conserved R’’-L’’ and L’-R’ regions on the top and bottom strands. Below, the secondary structure of the *attC*_*aadB*_ bottom strand illustrates the pairing of R’’/R’ and L’’/L’ to form the R and L boxes, exposing the extrahelical bases (EHBs) essential for IntI recognition. The predicted structure was determined by the RNAfold online interface (http://rna.tbi.univie.ac.at/cgi-bin/RNAWebSuite/RNAfold.cgi). **(c)** Genetic architecture of unusual Δ*attI*-type gene cassettes. The *bla*_OXA-10_ and *ybeA* genes are depicted as representatives of unusual Δ*attI1*-type and Δ*attI2*-type gene cassettes, respectively. The expanded sequence views detail the recombination sites (position -11), in light red for *attI1*, and cyan for *attI2*. Underlines indicate the possible extension of the Δ*attI1* and Δ*attI2* sites considered for this study, while red nucleotides highlight the gene cassette stop codons.

One of the most widespread molecular mechanisms that allows Gram-negative bacteria to adapt to diverse ecological niches is the integron/gene cassette system (Fig. 1A)^1–5^. As a main feature, the integron’s integrases mediate the site-specific recombination of at least three types of DNA target sites with distinct genetic structures and requirements: the *attI* site that usually lies adjacent to the *intI* gene, the *attC* site found in canonical gene cassettes (Fig. 1A), and the more recently described *attG* sites which exhibit significant sequence and structural divergence from classical *att* sites and serve as recombination sites for insertion of gene cassettes into bacterial genomes^4,6–10^. When modifying habitats and/or under stress conditions, bacteria carrying IntI can reorganize genes in the form of gene cassettes through site-specific recombination events. The acquisition of gene cassettes encoding beneficial proteins into the stable genetic platform of integrons (Fig. 1A) ensures their selection and enhances the host bacteria’s survival. Notably, different integron’s integrases that share less than 40% amino acid identity can also recognize the same *attC* site^5^. Their expression is driven by a Pc promoter located within or very close to the *intI* gene^11^. This system provides a rapid adaptive advantage in response to environmental changes.

The *attC* sites display significant sequence and length variation (ranging from 57 to 141 bp) among different gene cassettes but maintain a conserved structural organization consisting of two regions of inverted homology, R’’-L’’ and L’-R’, flanking a variable-length central region (Fig. 1B)^12–15^. Complementarity between these regions facilitates the formation of the characteristic *attC* secondary structure, which is essential to be maintained, since any variation would modify the site-specific recombination process (Fig. 1B)^12,15,16^. Despite the absence of defined consensus sequences within L’’ and L’ regions, their pairing produces extrahelical bases (EHBs) that are required for recombination^15,17^. At the left-hand end, the R’’ site (also called inverse core site, ICS) is formed by the consensus sequence 5’-RYYYAAC-3’ (Y: pyrimidine; R: purine), while at the right-hand end lies the R’ site (also called core site, CS) following the consensus sequence 5’-GTTRRRY-3’ (Fig. 1B) (reviewed in Escudero et al., 2015)^18^. The recombination crossover point is located between the first and second nucleotides of the R’ site, of the bottom strand (Fig. 1B)^8,13^.

The paradigmatic *attI1* site of class 1 integrons is 65 base pairs (bp) long (or *attI1*_− 56_ relative to the G crossover point) and comprises the L box 5’-CCCTAAA-3’ and the R box, which follows the consensus sequence 5’-GTTRRRY-3’, with two further direct repeats (DR1 and DR2) located upstream of these boxes (Fig. 1A)^19–24^. It has been demonstrated using biochemical in vitro assays that IntI1 recombinase binds to four regions of double-stranded (ds) *attI1*^19,25^, two of them belonging to the L and R boxes and two to the DR1 and DR2^19,20^. The specific structural elements and binding sites characterized for *attI1* have not yet been defined for the *attI2* site of class 2 integrons. Consequently, some studies have considered the *attI2* region to span approximately 311 bp, extending from the translation initiation codon of the *intI2* gene to the recombination crossover point^11,37^ (Fig. 1A). Recently, Vorobevskaia et al. (2024) confirmed by an optical tweezers force-spectroscopy assay that four IntI1 molecules and two recombination sites form a macromolecular synaptic complex, which is key to drive the recombination process^26^. A strong correlation between recombination efficiency and mechanical stability of the synaptic complex was found, suggesting sequence-dependent regulation through complex stabilization^26^.

Class 1 integrons are very successful in the acquisition, abundance, maintenance and spread of antimicrobial resistance gene cassettes (ARCs) among Gram-negative bacilli isolated from clinical samples. Their variable regions can accumulate up to eight ARCs (Accession number: LC333379) and even ten gene cassettes (Accession number: KC170993) in multiple combinations encoding antimicrobial resistance mechanisms (ARM) for almost all families of antibiotics. Noteworthy, it is very rare to find the same ARC duplicated in tandem within the variable region of mobile integrons (MIs). Class 2 and 3 integrons that are also MIs, share only a few of the wide pool of ARCs found in clinical isolates^27^. The *attC* sites of ARCs described in the hospital environment are highly heterogeneous among themselves^13^. However, each specific gene cassette is consistently associated with its particular *attC* site, and variations from this pattern may indicate unusual recombination events^3^. This contrasts sharply with the homogeneous *attC* sites found in sedentary chromosomal integrons (SCIs) of diverse bacterial species^28,29^.

Some gene cassettes including several ARCs containing modifications in the *attC* site recognition sequences have been identified^3,30^. Many of these atypical gene cassettes, called “unusual” for convenience in this article, have not been identified as such in GenBank Database nor in the literature. These modifications may have implications both at the recombination level with integrases as well as in downstream gene cassette expression. In a previous study, Partridge et al., 2009^3^ identified three types of unusual gene cassettes formed by: i) gene cassettes that have the *attC* site truncated by deletions where only a few characteristic sequences such as the R’’ of the original *attC* site are recognizable; ii) gene cassettes where the *attC* site is replaced by a deletion of the *attI* site which we call unusual Δ*attI*-type gene cassettes in this study (Fig. 1C), and iii) gene cassettes containing *attC* sites disrupted by insertion sequences or class C group II-attC introns.

Recombination frequencies mediated by IntI1 have been previously evaluated for several canonical gene cassettes with different genetic architectures. These studies examined *attI1*-ORF-*attC* and *attC*-ORF-*attC* arrangements in excision/circularization/insertion mechanisms, as well as *attI1*x*attI1*, *attI1*x*attC* and *attC*x*attC* pathways for co-integrate formation and resolution^10,12,22,23,31–34^. However, scarce data has been reported regarding recombination of the naturally found unusual Δ*attI*-type gene cassettes^35^. Previously, it has been suggested by Partridge et al., (2000), that an uncommon recombination crossover point between the L of the *attI1* site and the R’’ in the *attC* site would give rise to a circularized gene cassette containing the unusual Δ*attI* site^23^.

An additional uncommon finding in integron arrays is the presence of canonical duplicated gene cassettes, such as those exhibiting *attC*_ORF_-ORF-*attC*_ORF_ genetic architectures (see below). The low frequency of these gene cassette arrangements in tandem suggests active mechanisms suppressing their formation or maintenance.

In this study, we identified clinical class 1 and 2 integrons as the most important representatives of MIs harbouring Δ*attI* sites (Fig. 1C). We selected canonical and unusual Δ*attI-*type gene cassettes from our integron´s collection for recombination studies; particularly we studied the *aadB* gene cassette (also named *ant(2´´)-I*) spanning 534 bp and conferring resistance to gentamicin, tobramycin and kanamycin. The unusual Δ*attI*-type gene cassettes are characterized by the replacement of the canonical *attC* site with a partial *attI1* or *attI2* site, generating the *attI*-ORF*-*Δ*attI* or *attC*-ORF*-*Δ*attI* genetic architectures. We found by functional analysis that unusual *attI1-aadB-*Δ*attI1*_− 11_ (positions are relative to the G’ insertion site in the *attI*) as well as *attC*_*aadA1*_-*aadB-*Δ*attI2*_− 238_ and *attC*_*aadA1*_-*ybeA-*Δ*attI2*_− 11_ gene cassettes serve as active functional substrates for IntI1 site-specific recombination. On the other hand, to further elucidate the mechanisms preventing duplication of gene cassettes we constructed the *attC*_*aadB*_-*aadB*-*attC*_*aadB*_ genetic architecture. Surprisingly, the recombination frequencies mediated by IntI1 were substantially higher than canonical *aadB* gene cassettes (*attI1*-*aadB*-*attC*_*aadB*_ and *attC*_*sat2*_-*aadB*-*attC*_*aadB*_) reaching values of up to 97% of excision. These results suggest a scenario where canonical gene cassettes undergo near-instantaneous excision upon insertion. In addition, the unusual *attI1*-*aadB*-*attI1* gene cassette had also 100% of excision mediated by IntI1 in our biological model without antibiotic pressure. These results contribute to unravel how evolutionary factors influencing integron recombination selected over time, amongst multiple features, two recombination sites, *attC* and *attI*, and to lesser extent Δ*attI* sites, to benefit a trade-off between both stability and diversity of arrays of gene cassettes within the variable region of class 1 and class 2 integrons.

## Results

### Identification of unusual Δ*attI*-type gene cassettes

Scarce data concerning gene cassettes with the *attC* site replaced by a deletion of the *attI* site (Δ*attI*-type) have been previously reported in class 1 and 2 integrons^3,30,35^. Such unusual Δ*attI*-type gene cassettes result in the *attI*-ORF-Δ*attI* or *attC*-ORF-Δ*attI* genetic architectures^35–38^.

**Fig. 2:**
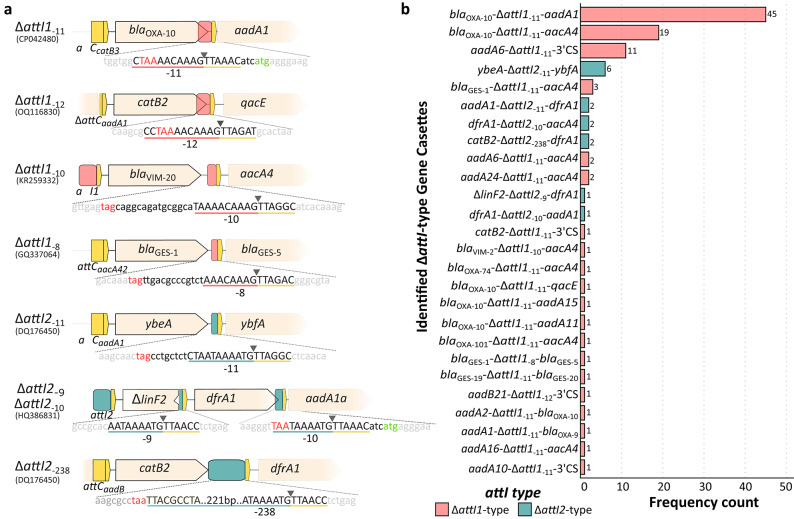
Genetic diversity and prevalence of unusual Δ*attI*-type gene cassettes. **(a)** Schematic representation of representative unusual Δ*attI*-type gene cassette arrangements identified in this study. The diagrams depict the gene cassettes (arrow boxes) and their associated recombination sites, where truncated Δ*attI1* and Δ*attI2* sites are colored in light red and cyan, respectively, and *attC* sites are shown in yellow. Representative GenBank accession numbers are listed on the left. Expanded nucleotide sequences detail the recombination region; uppercase letters denote recombination site sequences, including the R’’ of the downstream gene cassette. Red nucleotides highlight the gene cassette stop codons, and green nucleotides indicate the start codons of the downstream gene. Grey arrowheads mark the specific recombination crossover point, and the underlines and numbers indicate the extent of the *attI* sequence relative to this point (e.g., -11). **(b)** Frequency distribution of the 26 unique unusual Δ*attI*-type gene cassette arrays identified in the analyzed databases. The bar chart ranks the arrays by abundance, color-coded to distinguish between those derived from *attI1* (light red) and *attI2* (cyan).

By bioinformatics analysis of complete class 1 (from publicly shared data of T. Jové) and class 2 (from the INTEGRALL database) integrons, we confirmed that unusual Δ*attI*-type gene cassettes are not restricted to a specific class of integrons (Supplementary Table S1). Sequence alignment revealed a conserved minimum consensus sequence for these sites: 5’-AAACAAAGTTRRRY-3’ for Δ*attI1* and 5’-AATAAAATGTTRRRY-3’ for Δ*attI2* (Figs. 1C and 2A). These Δ*attI* sites contained variable-length sequences of *attI1* or *attI2* sites extending from this core sequence, as part of different unusual Δ*attI*-type gene cassettes (Supplementary Table S1, Fig. 2A).

We analyzed 1,685 class 1, and 57 class 2 complete integrons, identifying 96 and 14 Δ*attI1* and Δ*attI2* unusual Δ*attI*-type gene cassettes, respectively (Supplementary Table S1). A total of eight types of unusual Δ*attI*-type gene cassettes were identified (Fig. 2A). Four corresponded to the *attI1* site retaining fragments of 18 nt of the *attI1* site (Δ*attI1*_− 12_), 17 nt (Δ*attI1*_− 11_), 16 nt (Δ*attI1*_− 10_) and 14 nt (Δ*attI1*_− 8_) (Fig. 2A). The remaining four derived from the *attI2* site, with conserved segments of 17 nt (Δ*attI2*_− 11_), 16 nt (Δ*attI2*_− 10_), 15 nt (Δ*attI2*_− 9_), and 244 nt (Δ*attI*_− 238_) (Fig. 2). The most prevalent variants were Δ*attI1*_− 11_ sites (*n* = 90) and Δ*attI2*_− 11_ (*n* = 8) for class 1 and class 2 integrons, respectively (Fig. 2B). Also, an unusual gene cassette with the complete *attI1* site (65 bp) upstream and downstream of Δ*aacA4* (*attI1-*Δ*aacA4-attI1*) was found (FN554979.1).

Within class 1 integrons, the unusual *bla*_OXA−10_-Δ*attI1*_− 11_ gene cassette was the most widespread since it was present in 65 out of 96 variable regions of class 1 integrons, usually followed by *aadA*_type_ gene cassettes (Fig. 2B).

Concerning the unusual Δ*attI2-*type gene cassettes, the most frequently detected was *ybeA*-Δ*attI2*_− 11_ present in 6 out of 12 class 2 integrons, from which two were previously found as the *attC*_*aadA1*_-*ybeA*-Δ*attI2*_− 11_ in the widespread Tn*7*::In2-7 (*ybeA* was formerly reported by our group and others as *orfX*) (Fig. 2B)^30,37^.

Interestingly, two unusual Δ*attI2*-type gene cassettes were found within the variable region of two class 1 integrons (In251 and In2123) (Supplementary Table S1), revealing cross-class interchanges of unusual Δ*attI*-type gene cassettes between the variable regions of MIs.

### Gene cassettes selected for recombinational studies identified in clinical isolates

For experimental studies, we selected three unusual Δ*attI*-type gene cassettes from our laboratory collection (Table 1). The first, *attI1*-*aadB-*Δ*attI1*_− 11_ was found within the variable region of a class 1 integron from *Klebsiella pneumoniae* Kpn10 (2014, Ciudad Autónoma de Buenos Aires), the second, *attC*_*aadA1*_-*aadB-*Δ*attI2*_− 238_ originated from the variable region of a class 2 integron from *Acinetobacter baumannii* A230 (2022, Ciudad Autónoma de Buenos Aires) while the third, *attC*_*aadA1*_-*ybeA-*Δ*attI2*_− 11_ derived from another class 2 integron from *A. baumannii* A543 (2020, Ciudad Autónoma de Buenos Aires) (Fig. 4A). These unusual Δ*attI1*-type gene cassettes have the particularity that downstream of the stop codon of the *aadB* gene, and corresponding to a rearrangement at the R’’ site, the expected *attC* site has been replaced by a 17 bp (position − 11) DNA region with 100% identity to *attI1* (NC_028464), or by a 244 bp (position − 238) DNA region with 100% identity to the *attI2* site (DQ176450), respectively (Fig. 4A). In the unusual Δ*attI2*-type gene cassette downstream of the stop codon of the *ybeA* gene and, corresponding to a rearrangement at the R’’ site, the expected *attC* site has been replaced by a 17 bp (position − 11) DNA region with 100% identity to *attI2* (DQ176450) (Fig. 4A).

**Table 1:** Features of plasmids used for recombination assays. S: fragment cloned on the same orientation of the primer or promoter mentioned. AS: fragment cloned on the opposite orientation of the primer mentioned. Cm^R^, Amp^R^ and Kn^R^: indicate resistance to chloramphenicol, ampicillin and kanamycin, respectively.

Plasmids	Gene cassette array or relevant genetic features	Reference	Accession number
Vectors			
pACYC184	Cloning vector (Cm^r^), p15a replication origin	New England Biolabs	X06403.1
pCR2.1 TOPO	Cloning vector (Amp^R^ Kn^R^), regulation with *lac* y *T7* promoters, ColE1 replication origin	Invitrogen	-
pAO	oriT R388 (pACYC184::oriT AS to pACYC5’ Cl^R^)	This study	-
**Intermediate Constructs**			
pC1AS	*attI1* (pCR2.1 TOPO::*attI1* AS to Plac Amp^R^ Kn^R^)	This study	-
pCpoly AS	*Polylinker::nde*I (pCR2.1TOPO::polylinker::*nde*I AS to Plac Amp^R^ Kn^R^)	This study	-
pLQ3poly	*intI1-polylinker::nde*I (pLQ369::*polylinker*::*nde*I AS to Plac Amp^R^)	This study	-
pC1Bc	*attI1-aadB-attC* (pCR2.1 TOPO::*attI1-aadB-attC* AS to Plac Amp^R^ Kn^R^)	This study	-
pCcBc	*attC-aadB-attC* (pCR2.1 TOPO::*attC*_*sat2*_ *-aadB-attC* AS to Plac Amp^R^ Kn^R^)	This study	-
pC1BΔc	*attI1-aadB-ΔattC* (pCR2.1 TOPO::*attI1-aadB-ΔattC* AS al Plac Amp^R^ Kn^R^)	This study	-
pCORFX_− 11_	*attC*_*aadA1*_-*ybeA*-Δ*attI2*_− 11_ (pCR2.1 TOPO::*attC*_*aadA1*_-*ybeA*-Δ*attI2*_− 11_ AS to Plac Amp^R^ Kn^R^)	This study	-
pC1B_− 11_	*attI1-aadB–*Δ*attI1*_− 11_ (pCR2.1 TOPO::*attI1-aadB–*Δ*attI1*_− 11_ AS to Plac Amp^R^ Kn^R^)	This study	-
pC*attI2*_− 238_	*attC*_*aadA1*_*-aadB-*Δ*attI2*_− 238_ (pCR2.1 TOPO::::*attC*_*aadA1*_*-aadB-*Δ*attI2*_− 238_ AS to Plac Amp^R^ Kn^R^)	This study	-
**IntI1-expressing plasmids**			
pLQ369	*intI1* (pMalC2x::*intI1* AS to M13R Amp^R^)	Gravel et al., 1998	-
pMI1-1	*intI1-attI1* (pLQ3poly::*attI1 –attI1* S to M13F- Amp^R^), Derived from pLQ369	This study	PV877221
**Final Recombinant Plasmids**			
pORFX_− 11_	*attC*_*aadA1*_-*ybeA*-Δ*attI2*_− 11_ (pACYC::*attC*_*aadA1*_-*ybeA*-Δ*attI2*_− 11_ S to pACYC5’ Cl^R^)	This study (template: *A. baumannii* A543)	PX206334
pAO1Bc	*attI1-aadB-attC*_*aadB*_ (pACYC::oriT*-attI1-aadB-attC* S to pACYC5’ Cl^R^)	This study (template: *S. marcescens* sm404)	PX257573
pAO1BΔc	*attI1-aadB-ΔattC*_*aadB*_ (pACYC::oriT*-attI1-aadB-ΔattC* S to pACYC5’ Cl^R^)	This study (mutated from pAO1Bc)	PX257573
pAO1B_− 11_	*attI1-aadB–*Δ*attI1*_− 11_ (pACYC::oriT-*attI1-aadB–*Δ*attI1*_− 11_ S to pACYC5’ Cl^R^)	This study (template: *K. pneumoniae* Kpn10)	PX206332
paadB-*attI2*_− 238_	*attC*_*aadA1*_*-aadB-*Δ*attI2*_− 238_ (pACYC::*attC*_*aadA1*_*-aadB-*Δ*attI2*_− 238_ S to pACYC5’ Cl^R^)	This study (template: *A. baumannii* A230)	PX206333
pAcBc	*attC*_*sat2*_*-aadB-attC*_*aadB*_ (pACYC184::*attC*_*sat2*_*-aadB-attC* S to pACYC5’ Cl^R^)	This study (template: *A. baumannii* A230)	PX206335
pAO1B1	*attI1-aadB-attI1* (pACYC::oriT*-attI1-aadB-attI1* S to pACYC5’ Cl^R^)	This study (synthetic structure)	-
pACA3	*attC*_*aadB*_*-aadB-attC*_*aadB*_ (pACYC184::*attC*_*sat2*_*-aadB-attC* S to pACYC5’ Cl^R^)	This study (synthetic structure)	-

In addition, two canonical gene cassettes were cloned: *attC*_*sat2*_*-aadB-attC*_*aadB*_ found in the variable region of a class 2 integron from *A. baumannii* A203 (2020, Ciudad Autónoma de Buenos Aires) and *attI1-aadB-attC*_*aadB*_ found in the variable region of a class 1 integron from *Serratia marcescens* sm404 (2002, Ciudad Autónoma de Buenos Aires) (Table 1).

**Fig. 3:**
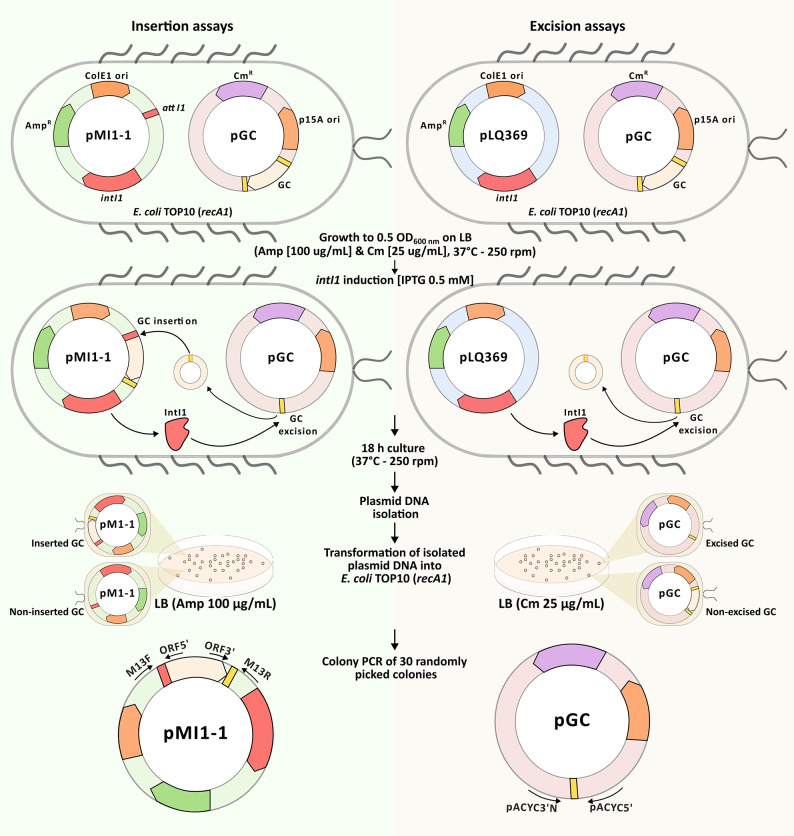
Schematic representation of the *in vivo* recombination assays. The experimental design employs a co-transformation biological model in *E. coli* TOP10 (*recA1*) to evaluate IntI1-mediated recombination frequencies. Left: In insertion assays, the recipient cells containing the pMI1-1 plasmid (Amp^R^), harboring both the *intI1* gene and the *attI1* recombination site, are co-transformed with the gene cassette-carrying plasmid pGC (Cm^R^). Following growth and IPTG induction of *intI1*, plasmid DNA is isolated and transformed into fresh competent cells. Selection is performed on LB agar with ampicillin (100 µg/mL) to recover the pMI1-1 population, and insertion events are detected by colony PCR using M13F and M13R primers combined with ORF specific primers. Right: In excision assays, the cells containing the pLQ369 plasmid (Amp^R^), which expresses *intI1* but lacks the *attI1* site, are co-transformed with pGC plasmid. After the *intI1* induction with ITPG, plasmid DNA is isolated and transformed into fresh competent cells under chloramphenicol (25 µg/mL) selection to recover the pGC population. Excision events are identified by PCR using specific primers (pACYC3’N and pACYC5’) to detect the size reduction associated with gene cassette loss.

### Recombination mediated by IntI1 of unusual Δ*attI*-type *aadB* gene cassettes

**Fig. 4:**
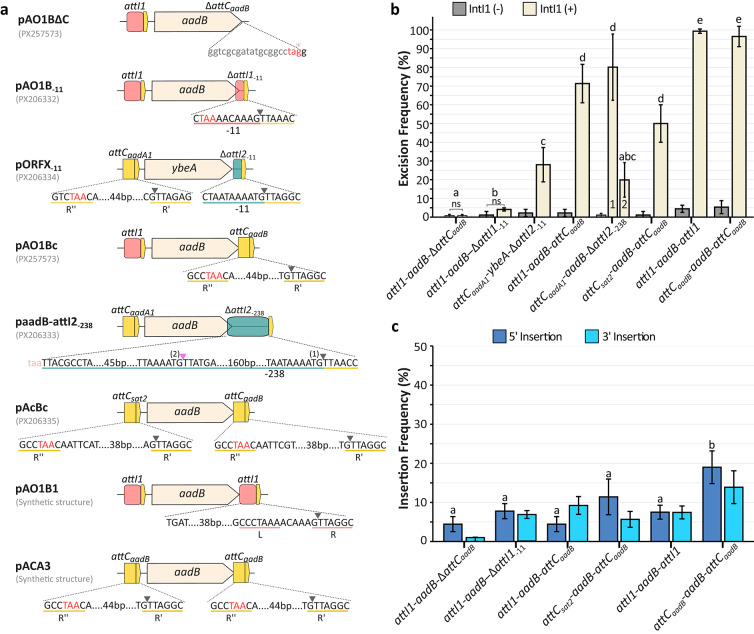
Gene cassette architectures and IntI1-mediated recombination frequencies. **(a)** Schematic representation of the recombinant constructs used to test the functionality of diverse gene cassette arrangements. Plasmid names and GenBank accession numbers are listed on the left. Expanded nucleotide sequences detail the recombination sites, where uppercase letters denote conserved motifs and grey arrowheads mark the canonical recombination crossover point (G). In the paadB-attI2_-238_ construct, an additional pink arrowhead (2) indicates a secondary, atypical recombination site identified in this study alongside the canonical site (1). The pAO1BΔC construct contains a mutated R’’ site (indicated in red) to serve as a negative control for excision. **(b**) IntI1-mediated excision frequencies. Yellow bars represent the excision frequency in the presence of IntI1, while grey bars indicate the IntI1-negative control. Notably, for the *attC*_*aadA1*_-*aadB*-Δ*attI2*_-238_ construct, the bars labeled 1 and 2 correspond to the excision events occurring at the primary and secondary cut sites shown in panel (a), respectively. All substrates showed significant excision activity compared to their respective intI1-negative controls (*p* < 0.05), except *attI1*-*aadB*-Δ*attC* and *attI1*-*aadB*-Δ*attI1*_-11_. Letters above bars indicate statistical groupings: architectures sharing the same letter are not significantly different, while different letters indicate significant differences (*p* < 0.05). **(c)** IntI1-mediated insertion frequencies. The bar chart displays the frequency of gene cassette capture into the *attI1* site of pMI1-1, differentiated by insertion orientation: dark blue bars represent insertion at the 5’ end, and light blue bars represent insertion at the 3’ end. Letters indicate statistical groupings (*p* < 0.05). For 5’ insertion: group “a” includes most genetic architectures, while group “b” contains only *attC*_*aadB*_-*aadB*-*attC*_*aadB*_ architecture. Data represents the mean ± SD of at least three independent biological replicates (*n*=3). Statistical significance was determined using pairwise Welch’s t-tests.

The principles of the *in vivo* recombination assays mediated by IntI1 are schematically illustrated in Fig. 3. This biological model employs a co-transformation assay with two plasmids: one containing the canonical or unusual Δ*attI*-type gene cassettes (e.g., *attI1*-*aadB*-*attC*_*aadB*_ and *attI1*-*aadB*-Δ*attI1*_− 11_) (pGC) and another harboring the inducible *intI1* gene (with or without the *attI1* recombination site for the insertion or excision assays, respectively). Each assay was performed with at least three biological replicates. Recombination assays showed that unusual *attI1-aadB-*Δ*attI1*_− 11_ gene cassette —as previously found for *attC*_*aacA4*_*-aadA1-*Δ*attI1*_− 11_ from our laboratory^35^— had a lower IntI1-mediated excision efficiency compared to canonical gene cassettes (*attI1-aadB-attC*_*aadB*_ and *attC*_*sat2*_*-aadB-attC*_*aadB*_) (Fig. 4B, Supplementary Table S2). On the other hand, *in vivo* recombination assays showed that this unusual Δ*attI1*-type gene cassette retains a similar capacity of canonical gene cassettes to be recognized and inserted in the *attI1* site (Fig. 4B, Supplementary Table S2). Sequence analysis of amplicons confirmed that the insertion of *attI1-aadB-*Δ*attI1*_− 11_ into the *attI1* site and the circularization of *attI1-aadB-*Δ*attI1*_− 11_ was by a site-specific recombination event mediated by IntI1. Unexpectedly, the excision frequency of *attC*_*aadA1*_-*ybeA-*Δ*attI2*_− 11_ was similar to that of other canonical gene cassettes (Fig. 4B, Supplementary Table S2). Sequence analysis of amplicons confirmed that the excision mediated by IntI1 of *attC*_*aadA1*_-*ybeA-*Δ*attI2*_− 11_ occurred by a site-specific recombination event. The *attC*_*aadA1*_-*aadB-*Δ*attI2*_− 238_ also proved to be an efficient substrate for IntI1-mediated recombination (Fig. 4B, Supplementary Table S2). Specific primers with divergent orientation in *attC*_*aadA1*_*-aadB-*Δ*attI2*_− 238_ were designed to detect the recombination site generated after the excision. Remarkably, two amplification products were obtained from this reaction, one of them of 450 bp and the other of 250 bp. Sequence analysis of both products revealed that two different sites can be recognized by IntI1 to mediate the excision (Fig. 4, Supplementary Table S2). One site corresponded to the G of the previously proposed crossover point 5’-G’TTAACC-3’ in the *attI2* of class 2 integrons^39,40^. The other recombination site, the one found in the 250 bp amplification product, revealed that in this case IntI1 recognized the G of the 5’-G’TTATGA-3’ sequence localized 62 bp downstream from the 5’-TAA-3’ of the *aadB* gene (Fig. 4A). In this event, IntI1 recognized an atypical core site to mediate the gene cassette excision. The excision frequencies were 80% and 20%, respectively, for each target (Fig. 4B, Supplementary Table S2). Taken together, these results demonstrate that unusual Δ*attI*-type gene cassettes are functional and recognizable as IntI1 substrates for excision and even for insertion.

### *attI1-aadB-attI1* and *attC*_*aadB*_-*aadB*-*attC*_*aadB*_ are the most efficient genetic architectures for site-specific recombination mediated by IntI1

To determine the effect of the complete *attI1* site instead of the Δ*attI1*_− 11_, we constructed *attI1-aadB-attI1* (Supplementary Table S1, Fig. 4A) as previously found for *attI1-*Δ*aacA4-attI1* (FN554979.1). Noteworthy, excision frequency mediated by IntI1 of *attI1-aadB-attI1* was 100% by using the expected site-specific recombination sites (Fig. 4B, Supplementary Table S2). Sequence analysis of the amplification products from the in vivo recombination assay for *attI1-aadB-attI1* as template revealed site-specific recombination of the circularized intermediate and insertion into the *attI1* site. Based on these results where the same *att* is upstream and downstream of the *aadB* ORF, and since the same *attC* site upstream and downstream of an ORF was not previously tested in recombinational studies, we analyzed the *attC*_*aadB*_-*aadB*-*attC*_*aadB*_ in recombination assays with IntI1 in our biological model of co-transformation without selection pressure with gentamicin. Besides, this genetic architecture is uncommon since the same gene cassette is not usually found one after the other in tandem arrays within the variable region of class 1 nor in class 2 integrons (Supplementary Table S3). Our bioinformatics analysis identified that only 28 out of 1,685 class 1 integrons have gene cassettes in tandem (Supplementary Table S3) showing limited dissemination to date (Data not shown).

Consistent with the previous result, the excision frequency for *attC*_*aadB*_-*aadB*-*attC*_*aadB*_ mediated by IntI1 was significantly higher than canonical *attI1*-*aadB*-*attC*_*aadB*_ and *attC*_*sat2*_-*aadB*-*attC*_*aadB*_ genetic architectures reaching values of 97% (Fig. 4B, Supplementary Table S2). Since selection pressure was applied exclusively to the plasmid backbone (Cm^R^), the observed 97% excision frequency highlights the natural tendency of these tandem arrays to resolve into single gene cassette copies, at least when a duplicated gene dosage is not required for survival. Also,* in vivo* recombination assays showed that unusual Δ*attI*-type gene cassette functions as a competent substrate for insertion in the *attI1* site with values equal or even higher than canonical gene cassettes (Fig. 4B, Supplementary Table S2). Our results align with previous findings demonstrating that gene cassettes whose upstream recombination site is an *attC* (such as *attC*_*sat2*_*-aadB-attC*_*aadB*_ and *attC*_*aadB*_*-aadB-attC*_*aadB*_) are better substrates for insertion into the *attI1* site than gene cassettes where the *attI1* is upstream the ORF (*attI1*-*aadB*-*attC*_*aadB*_)^17^.

As a control, we constructed the *attI1*-*aadB*-Δ*attC*_*aadB*_ genetic structure, where the R’’ of the *attC* site was point-modified to inactivate it and to preserve the ORF (5’-GCCTAAC-3’ → 5’-GCCTAGG-3’) and the rest of the *attC* site was removed yielding only one canonical target site for IntI1 (the complete *attI1* site upstream *aadB*) (Fig. 4A). This genetic structure was not excised in recombination assays with IntI1 (Fig. 4B, Supplementary Table S2).

Our results showed that a balance between different recombination sites, including *attI*, Δ*attI* and different *attC* sites in the genetic architecture of canonical and unusual Δ*attI*-type gene cassettes contribute to the diversity of arrays in the variable region of MIs.

**Fig. 5:**
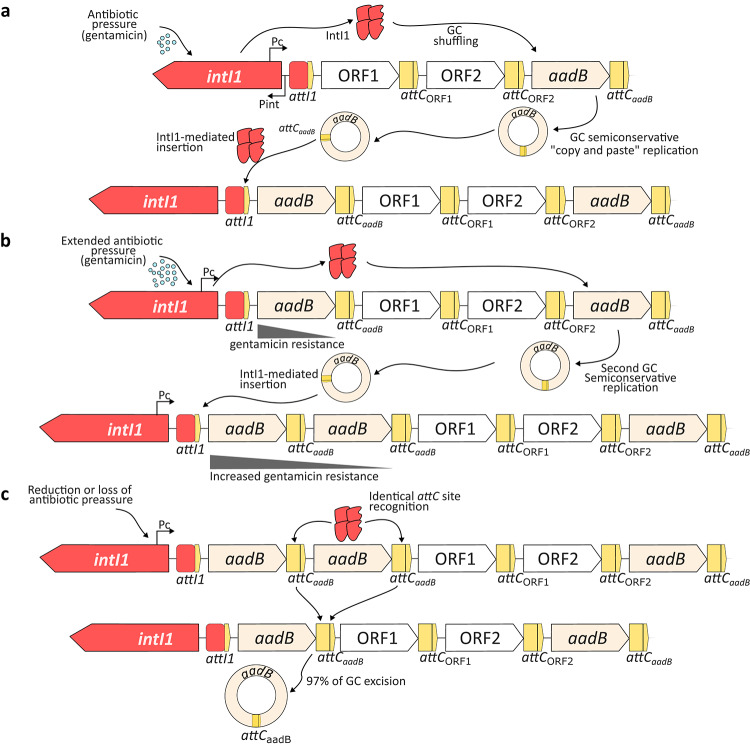
Proposed biological model for the transient formation and resolution of tandem gene cassette arrays under varying selective pressures. **(a)** Under initial antibiotic pressure (e.g., gentamicin), the bacterial SOS response triggers *intI1* expression. The integrase mediates the excision and semiconservative “copy and paste” replication of a distal *aadB* cassette, facilitating its capture at the primary *attI1* recombination site to enhance expression via the Pc promoter. **(b)** Under extended antibiotic pressure, the continued recruitment of *aadB* copies leads to the formation of a tandem gene cassette array (*attC*_*aadB*_*-aadB-attC*_*aadB*_), resulting in increased gene dosage and higher antimicrobial resistance levels. **(c)** Upon the cessation of antibiotic pressure, the tandem arrangement proves structurally unstable. The presence of identical *attC* sites flanking the *aadB* ORF serves as a highly efficient substrate for IntI1-mediated recombination. Consistent with the experimental data from this study, which showed a 97% excision frequency for this genetic architecture, the duplicated gene cassette is rapidly excised. This high-frequency resolution mechanism prevents the accumulation of duplicated gene cassettes, thereby favoring the maintenance of genetic diversity within the integron variable region.

## Discussion

There is extensive documentation regarding the molecular and biological basis of the integron/gene cassette system at many levels including dissemination^41–46^, functionality^11,16,19,23,47,48^, mechanisms involved in site-specific recombination^7,10,15,19,20,31,37,40,49–51^, environmental, clinical dispersal^1,52,53^ and evolution^29,54,55^, among others.

However, several relevant knowledge gaps remain in the universe of integrons, such as the molecular genesis of gene cassettes, and the discrepancy between the constant maximum amount of 8 or 10 gene cassettes typically found in class 1 integrons recovered from humans, and the more than 100 gene cassettes observed in many SCIs. Further research is also needed to determine the efficiency of gene cassette shuffling within integrons, the functions of many gene cassettes that have not yet been characterized and the role of selection pressures from various substances (e.g., antibiotics, heavy metals, etc.) on MIs and their subsequent impact on environmental and human bacterial populations that also remain unclear. Finally, the functionality of unusual gene cassettes still requires comprehensive understanding.

While site-specific recombination of canonical gene cassettes is recognized to involve specific requirements for the *attI* and *attC* sites that impact on the excision/insertion frequencies mediated by integron’s integrases^15,17,50^, the extent of the functionality nor the origin of Δ*attI* sites remain largely unknown. A possible explanation for the genesis of Δ*attI1*-type gene cassettes, at least for the most prevalent variant, the Δ*attI1*_− 11_ site, is that recombination between the L-box of the *attI1* site and the R’’ in the *attC* site would give rise in a single step to a circularized gene cassette containing the Δ*attI1*_− 11_ site, as previously proposed by Partridge et al., 2000. Furthermore, when we analyzed the *attC* site secondary structure of the most widely disseminated Δ*attI*-type gene cassettes, such as *bla*_OXA−10_ and *aadA6*, we found that both lack of at least one of the two specific EHBs required for the canonical single-stranded folding recognition described by MacDonald et al. (2006) (Supplementary Fig. S3). Within this scenario, it is likely that this structural deviation impedes the formation of the highly efficient single-stranded synaptic complex, thereby biasing the reaction towards the ancestral double-stranded recombination pathway identified by Escudero et al. (2016)^10^, driving the recombination between the L-box of the *attI1* site and the R-box in the *attC* site and, therefore, giving rise to the formation of the Δ*attI1*_− 11_ sites. Although some possible explanations concerning the genesis of Δ*attI* sites are presented, it is uncertain if this hypothesis is replicable for other Δ*attI1* or Δ*attI2* sites. Future experimental studies will be required to elucidate the molecular mechanism of the steps involved in their creation.

Bioinformatics analysis across different class 1 and 2 integron databases identified eight types of unusual Δ*attI*-type gene cassettes, revealing fragments of varying sizes corresponding to Δ*attI1* or Δ*attI2* sites within different gene cassette arrays. The most prevalent variants of Δ*attI* sites were Δ*attI1*_− 11_ (*n* = 90) and Δ*attI2*_− 11_ (*n* = 8). These variants correspond to 26 unusual Δ*attI*-type gene cassettes conferring resistance to four different families of antibiotics. This finding highlights their critical role as an important reservoir of ARCs that actively disseminate mobile ARM. The unusual Δ*attI*-type gene cassette *bla*_OXA−10_-Δ*attI1*_− 11_ was the most frequently detected among these.

It is well known that the efficiency of recombination with different *attC* sites and *attI* sites varies several orders of magnitude^10,15,17,50^; the underlying explanation is likely being connected to several factors^15–17^. Based on this, the study of unusual Δ*attI-*type gene cassettes become relevant because they represent another echelon in the entire biology of the integron/gene cassette system.

In the present study, we focused on several unusual Δ*attI-*type gene cassettes from both class 1 and 2 integrons identified in our laboratory (Fig. 4). These gene cassettes which possess the Δ*attI* site located downstream of the ORF such as *attI1*-*aadB*-Δ*attI1*_− 11_, *attC*_*aadA1*_-*aadB*-Δ*attI2*_− 238_, and *attC*_*aadA1*_-*ybeA*-Δ*attI2*_− 11_ were excised at different rates (4%, 20/80% and 28%, respectively). Unexpectedly, IntI1 activity was more functional over Δ*attI2*_− 11_ than Δ*attI1*_− 11_. This suggests that unusual Δ*attI2* gene cassettes found in the variable region of class 2 integrons may be mobilized and even originated by IntI1. This is plausible since IntI2 is usually not functional in clinical isolates due to a premature stop codon at position 169^37^. Supporting this hypothesis, our *in silico* analysis identified two class 1 integrons containing both Δ*attI1* and two Δ*attI2* sites (MT813046 and HQ386834; Supplementary Table S1), representing potential instances of cross-class recombination mediated by IntI1.

Interestingly, previous results from our laboratory showed that canonical Δ*attI*_− 11_-*aac4*-*attC*_*aac4*_ and Δ*attI*_− 11_-*bla*_OXA−9_-*attC*_*bla*OXA−9_ gene cassettes from Tn*1331* were not excisable by IntI1^35^, in agreement with the negative result of excision of canonical Δ*attI*_− 7_-*aacA4*-*attC*_*aacA4*_ gene cassette from Tn*1404* as described by Partridge et al. (2002)^56^. Optical tweezers force-spectroscopy assays will be crucial to probe the synaptic complex stability of these canonical gene cassettes harbouring Δ*attI* sites upstream of the ORF (which are non- excisable) as opposed to unusual Δ*attI-*type gene cassettes (which are excisable) that have the Δ*attI1* site downstream of the ORF, as previously done by Vorobevskaia et al. (2024)^26^ with other canonical gene cassettes.

Our results also showed that IntI1 has the faculty to recognize and mediate not only the excision of unusual Δ*attI-*type gene cassettes, but also their insertion in another complete *attI1* site (Fig. 4B). Partridge et al. (2000) showed that in co-integration assays, the *attI1* L and R boxes in the region named “simple site” (-25 + 32) alone are active at low levels in reactions with a complete *attI1* site (65 bp) being not as efficient in reactions with *attC* sites^23^. However, since in those studies the recombination assays were performed by the formation of co-integrates, their results cannot be extrapolated to our assays, in which the entire genetic architecture of the unusual Δ*attI-*type gene cassettes is investigated in insertion experiments into the *attI1* site (Fig. 4B). Our results for insertion of unusual Δ*attI-*type gene cassettes into the *attI1* site were similar to canonical gene cassettes (Supplementary Table S2). These findings, plus the diversity of arrays with Δ*attI-*type gene cassettes for *bla*_OXA−10_-Δ*attI*_− 11_ found in databases, support the full functionality and efficiency of unusual Δ*attI-*type gene cassettes in recombination assays suggesting their potential mobility and spread amongst integrons after their formation.

The obtained results and the recently described activity of the IntI1 to recognize *attG* sites as targets for insertion of gene cassettes into bacterial genomes^57^, highlight the remarkable plasticity exposed by IntI1 to recognize a broad range of substrates. All together, these findings support the hypothesis that IntI1 has many opportunities in nature to recombine with new targets and to recruit and disseminate new unusual Δ*attI-*type gene cassettes adding more advantages to bacteria to survive in hostile and changing environments.

As we identified another unusual Δ*attI-*type gene cassette with the genetic architecture *attI1-*Δ*aacA4-attI1* (FN554979.1), with the complete *attI1* site downstream of the ORF, we constructed a similar unusual gene cassette *attI1-aadB-attI1* for functional investigation. Although it is not the typical unusual Δ*attI-*type gene cassette as defined by Partridge et al. (2009) since the *attI1* site is complete, these genetic architectures are closely related. Noteworthy, excision frequency was 100%, evidencing that once inserted the unusual *attI1-aadB-attI1* gene cassette in an *attI1* site, it is immediately cleaved by IntI1. This extremely high frequency explains why it is rare to find this type of unusual *attI-*type gene cassette in the vast number of bacterial genomes that possess class 1 integrons.

Another knowledge gap concerns the molecular reasons underlying the uncommon finding of identical gene cassettes in tandem within the variable region of MIs, despite the mechanistic ability of integrons to duplicate gene cassettes. Our analysis of T. Jové’s dataset revealed that 28 out of 1,685 class 1 integrons contained such tandem arrangements (Supplementary Table S3). Most cases involved two gene cassette repeats, with one exceptional integron (AP024404) containing four consecutive *bla*_GES−24_ gene cassettes.

The unusualness of this arrangement was also evident in SCIs where, among the 143 fully assembled *Vibrio cholerae* RefSeq genomes deposited in GenBank, only one case of a gene cassette duplication was identified (Supplementary Table S3). In fact, this single instance involved the *dfrA31* gene cassette repeated three times in the *Vibrio cholerae* VC_hf7 strain (Supplementary Table S3).

Previous studies showed duplication and maintenance of some gene cassettes, such as *aadB*, but all experimental data were obtained under antibiotic pressure^48,58^. By using our biological model, the selection pressure was directed only to the plasmid backbone, allowing gene cassette mobilization without antibiotic pressure (Fig. 4).

We constructed the *attC*_*aadB*_-*aadB*-*attC*_*aadB*_ gene cassette to evaluate the role of the same *attC* (*attC*_*aadB*_ in our case) in the formation of tandem gene cassette arrays within the variable region of MIs. The IntI1-mediated excision frequency for the *attC*_*aadB*_-*aadB*-*attC*_*aadB*_ gene cassette reached values of 97% (Fig. 4B). This high rate shows that when this gene cassette is inserted, it is almost immediately excised, which contributes to the understanding of why duplicated gene cassettes are rarely observed within the variable region of MIs (Fig. 5). In addition to previous works that showed maintenance of duplicated gene cassettes under antibiotic pressure^48,58^, we cannot rule out that the finding of the *dfrA31* gene cassette in tandem in *V. cholerae* VC_hf7 strain is also due to antibiotic pressure, since treatment options to combat cholera include regimens with trimethoprim combined with sulfamethoxazole. Therefore, maintenance or loss of duplicated gene cassettes will depend on whether they are under selection pressure; other mechanisms such as homologous recombination can also play an underestimated and not yet studied role in the loss of duplicated gene cassettes.

Within the framework of this data, the circumstance by which the same gene cassette is not duplicated promotes the evolutionary selection of diverse gene cassette arrays within the variable region of MIs. Although it is unknown why class 1 and 2 integrons do not have more than ten gene cassettes in their variable region, the fact that the same gene cassette cannot be in tandem contributes to ensure the gene cassette diversity in a single MI. Therefore, the inability of gene cassettes to be duplicated favors the incorporation of new gene cassettes, thus enhancing genetic biodiversity and enriching the gene cassette reservoir by promoting broader dissemination of AMR in the hospital niche. Furthermore, it is likely that a balance exists between the need of genetic biodiversity and achieving high expression levels. This is because, in the face of a stress event (such as antibiotic pressure)^48,58^, duplicated gene cassettes can be maintained and allow the survival of host cells by increasing their expression (Fig. 5). Also, the fact that a single bacteria can harbor multiple MIs with the same or different gene cassette arrays, would contribute to maximize the expression of each gene cassette when the conditions require or favor it.

The evolutionary forces that shaped the integron/gene cassette system optimized its success in bacterial genomes. Over time, these forces must have selected excision and insertion frequencies that ensure both transcriptional stability of gene cassettes and adaptive responses to changing habitat pressures. In this scenario, the source of unusual Δ*attI-*type gene cassettes must add novel and unique advantages that also contribute to maintaining biodiversity within the variable region of MIs, and probably, also in SCIs. A trade-off between different recombination sites, including *attI*, Δ*attI* and different *attC* sites in the genetic architecture of canonical and unusual *attI-*type gene cassettes, is an important key for the diversity of arrays. Addressing these knowledge gaps is crucial for a comprehensive understanding of integron evolution and its implications for bacterial adaptation and the spread of mobile AMR.

## Materials and methods

### Bacterial strains

DNA from several clinical isolates from our strain collection (data not shown) was used as template for cloning and further studies (Table 1). The canonical gene cassettes *attI1*-*aadB*-*attC*_*aadB*_ and *attC*_*sat2*_-*aadB*-*attC*_*aadB*_ were obtained from *S. marcescens* sm404 (2002, Ciudad Autónoma de Buenos Aires) and *A. baumannii* A203 (2020, Ciudad Autónoma de Buenos Aires), respectively.

Three strains provided the unusual Δ*attI*-type gene cassettes for cloning and recombination assays: *K. pneumoniae* Kpn10 (2014, Ciudad Autónoma de Buenos Aires) with *attI1*-*aadB-*Δ*attI1*_− 11_, *A. baumannii* A543 (2020, Ciudad Autónoma de Buenos Aires) with *attC*_*aadA1*_-*ybeA-*Δ*attI2*_− 11_, and *A. baumannii* A230 strain (2022, Ciudad Autónoma de Buenos Aires) with *attC*_*aadA1*_-*aadB-ΔattI2*_− 238_.

*E. coli* TOP10 cells (Invitrogen) (F- *mcrA* Δ(*mrr-hsdRMS-mcrBC*) *φ80lacZ*Δ*M15* Δ*lacX74 deoR nupG recA1 araD139* Δ(*ara-leu*)7697 *galU galK rpsL*(*StrR*) *endA1 λ-*) were used as the recipient strain for plasmids, as well as for the recombination assays. The presence of the *recA1* allele ensures the strain is deficient in homologous recombination, ensuring that the observed recombination events are predominantly mediated by the site-specific integrase IntI1.

### Plasmids used for the *in vivo* recombination assays

The plasmid pMI1-1 containing the *attI1* recombination site and the *intI1* gene was constructed based on plasmid pLQ369^20^. Initially, the multiple cloning site from pCR2.1 TOPO was obtained by PCR amplification with primers M13F-NdeI and M13R-NdeI (Table 2) and then introduced into pLQ369 by digestion with *Nde*I restriction enzyme (New England Biolabs) and ligation with T4 DNA ligase (New England Biolabs) to obtain pLQ3poly. In a second step, the complete *attI1* recombination site was amplified with primers *attI1 Avr*II F and *attI1* (*qacE*) *Not*I R (Table 2) from *S. marcescens* SCH909 (GCF_015074945.1), cloned into pCR2.1 TOPO using the TOPO TA cloning kit (Invitrogen, CA) to generate pCRattI1, which was then used as substrate for subcloning the *attI1* site into the multiple cloning site of pLQ3poly to obtain pMI1-1. The whole pMI1-1 sequence was verified by Oxford Nanopore technology (Accession number: PV877221). Most of the plasmids used for in vivo recombination assays (pORFX_− 11_, paadB-*attI2*-_238_, pAcBc, pAO1Bc, pAO1B_− 11_, and pAO1BΔC) were constructed by PCR amplification of the canonical or unusual Δ*attI*-type gene cassettes from the total DNA of the strains mentioned above (Table 1) followed by cloning into pCR2.1-TOPO using the TOPO TA cloning kit (Invitrogen, CA) and subcloning into the pACYC184 plasmid backbone. The pORFX_− 11_, paadB-*attI2*-_238_ and pAcBc plasmids were generated by subcloning from the pCR2.1-TOPO based plasmids into pACYC184 using *Hind*III and *Bam*HI (New England Biolabs) and T4 DNA ligase (New England Biolabs). The pAO1Bc, pAO1B_− 11_ and pAO1BΔC plasmids were generated by subcloning from the pCR2.1-TOPO based plasmids into the pAO mobilizable plasmid, which is a pACYC184 derivative harboring the *ori*T from R388 plasmid. These subclonings were done by the digestion of the pCR2.1-TOPO based plasmids with *Spe*I and *Xba*I (New England Biolabs), and digesting the pAO plasmid with *Spe*I (New England Biolabs) and ligation with T4 DNA ligase (New England Biolabs) (Table 1). Particularly, the unusual *attI1*-*aadB*-*attI1* and the *attC*_*aadB*_-*aadB*-*attC*_*aadB*_ gene cassettes were synthesized into the commercial vector pMG-Kan (Km^R^) (Macrogen Inc., Korea) and subcloned into pAO and pACYC184 by digestion of both vectors with *Eco*RI and *Bam*HI. Each of these plasmids is based in the pACYC184 backbone harboring the canonical or the unusual Δ*attI-*type gene cassettes, which were co-transformed into *E. coli* TOP10 either with pLQ369 (*intI1*+, *attI1*-) for excision assays, or pMI-1 (*intI1*+, *attI1*+) for insertion assays.

**Table 2:** Primers used in the study.

Primers	Sequence (5’-3’)	Reference
pACYC184-5’	TGTAGCACCTGAAGTCAGCC	Gravel et al., 1998b
pACYC184-3´N	GTGATGTCGGCGATATAGGC	Ramírez MS PhD thesis, 2008
23´CS	TGGGCTGAGAGAGTGGT	Ramírez et al., 2005
3’CS New	AAGCAGACTTGACCTGATAG	This study
sulpro	GCCTGACGATGCGTGGA	Lévesque et al., 1995
*aadB* stop *Avr*II R	ATAAACCTAGGCCGCATATCGCGAC	This study
*attI1 Avr*II F	ATAAACCTAGGCGTTACGCCGTGGGTCG	This study
*attI1* (*qacE*) *Not*I R	AATATAAAGCGGCCGCTGCATCTAACTTTGTTTTAG	This study
ORITF	GTCCGTTTCATTCACTTGTAG	This study
M13F ndeI	CATATGGTAAAACGACGGCCAG	This study
M13R ndeI	CATATGCAGGAAACAGCTATGAC	This study
M13F	GTAAAACGACGGCCAG	Universal primer
M13R	CAGGAAACAGCTATGAC	Universal primer
aadBF3’	GATTACTTTTACTATGCCGATG	This study
aadBR5’	AAGAATCCATAGTCCAACTCC	This study

### *In vivo* recombination assays

To evaluate the IntI1-mediated frequencies of excision or insertion of canonical or unusual gene cassettes into the *attI1* recombination site, we carried out *in vivo* recombination assays by a biological model of co-transformation followed by PCR detection and confirmation by sequencing (Fig. 3). Briefly, each assay involved co-transformation of two plasmids into *E. coli* TOP10 were, one plasmid carried the genetic architecture to be analyzed (i.e., a canonical or an unusual Δ*attI-*type gene cassette) and the other contained the inducible *intI1* gene^20,34^ (with or without the *attI1* recombination site for the insertion or excision recombination assays, respectively). The antibiotic selection pressure was applied only directly to the plasmid backbones rather than the gene cassettes themselves. Remarkably, the PCR detection used in this technique allows us to accurately differentiate the excision, insertion at the 5’ and/or 3’ ends, co-integrates formation, and complete gene cassette insertion by the amplification of bands of a given molecular weight and sequencing confirmation.

The plasmids containing the different genetic structures listed in Table 1 were used to perform the in vivo recombination assays.

For the in vivo excision recombination assays *E. coli* TOP10 chemically competent cells were sequentially co-transformed with the plasmid harboring the chosen gene cassette architecture (pGC in Fig. 3, chloramphenicol resistant -Cm^R^-) and the pLQ369^20,34^ plasmid (ampicillin resistant -Amp^R^-), which contains the inducible *intI1* gene via heat shock. For insertion assays, pGC was co-transformed with the plasmid pMI1-1 (Amp^R^, expressing *intI1* and harbouring the *attI1* site) (Fig. 3). Co-transformant cells were selected by incubation at 37 °C in Luria Bertani (LB) agar supplemented with ampicillin [100 µg/mL] and chloramphenicol [25 µg/mL], overnight. A single co-transformant colony was picked and grown at 37 °C and 250 rpm in 5mL of LB broth supplemented with ampicillin [100 µg/mL] and chloramphenicol [25 µg/mL], for 18 h. Following this initial period, a 10% aliquot of the culture was transferred to a final volume of 5 ml of fresh LB broth supplemented with ampicillin [100 µg/mL] and chloramphenicol [25 µg/mL] and grown at 37 °C and 250 rpm to reach 0.5 OD_600nm_. At this point, the *intI1* expression (necessary for the IntI1-mediated recombination) was induced by the addition of 0.5 mM isopropyl-β-d-thiogalactopyranoside (IPTG) followed by 18 h incubation at 37 °C with agitation at 250 rpm^20^. After this, the recombination assay was stopped by the DNA extraction of the plasmid population using a Miniprep Kit (QIAprep Spin Miniprep Kit, Qiagen) in 50 µl of H_2_O. This DNA from the plasmid extraction was used as PCR template for detection and analysis of recombination events using appropriate primer pairs (Table 2; Fig. 3, Supplementary Fig. S1 & S2). At this step, the occurrence of recombination in this given assay was confirmed and then we proceeded with the frequency determination. For this, we used 2 µl of the isolated plasmid DNA to transform *E. coli* TOP10 chemically competent cells via heat shock. For excision frequency, 100 µl of the cell suspension was plated on LB plates supplemented with chloramphenicol [25 µg/mL] and incubated at 37 °C overnight, to select bacteria containing the native and/or recombinant pGC plasmids (Fig. 3). To determine insertion frequency, 100 µL of the cell suspension was plated on LB plates supplemented with ampicillin [100 µg/mL] and incubated at 37 °C overnight, to select bacteria containing the native and/or recombinant pMI1-1 plasmids (Fig. 3). In both cases, each replicate of the assay consisted of picking 30 colonies randomly distributed on the plate to carry out colony PCR with the selected set of primers. The number of colonies with positive bands of the specific size corresponding to the excision or insertion event were counted (Supplementary Fig. S1 & S2). Each recombination assay was performed at least three times in independent experiments for each genetic architecture to estimate the average of the frequencies shown (Fig. 4B).

To confirm the recombination events from both the excision and the insertion assays, the amplified PCR products were gel purified using the Wizard SV Gel and PCR clean-up System kit (Promega, USA) according to the manufacturer’s directions and both DNA strands were sequenced using an ABIPrism 3100 BioAnalyzer equipment.

As a positive control for both the excision and insertion recombination assays, parallel assays were carried out using the pAcBc plasmid in the same conditions described above. Also, as a negative control, parallel assays were performed using only the gene cassette harboring plasmid for each of the genetic architectures analyzed, without the IntI1-containing plasmids (pLQ369 or pMI1-1), to verify that the observed recombination events were primarily linked to the IntI1 integrase activity.

The entire co-transformation and induction process was performed in three independent biological replicates (*n* = 3) for all plasmids, except for pACA3 where six biological replicates (*n* = 6) were performed.

### Statistical analysis

IntI1-mediated frequencies of excision or insertion of canonical or unusual gene cassettes are expressed as the mean ± standard deviation (SD) of at least three independent biological replicates. Prior to analysis, data distribution and variance homogeneity were assessed. Given the sample size (*n* = 3) and the observed unequal variances between high-frequency and low-frequency recombination events, statistical significance was determined using pairwise Welch’s t-tests. In all cases, a *p*-value < 0.05 was considered statistically significant. All analyses were performed using R v4.5.1 (https://www.r-project.org/).

### Bioinformatics analysis

To identify and analyze unusual Δ*attI*-type gene cassettes, we compiled a set of integron sequences by first collecting accession numbers corresponding to a representative set of complete integrons. Class 1 integron accession numbers were obtained from publicly shared data by Thomas Jové (April 2025; https://annuel2.framapad.org/p/r.461a5f838700ae34c5d12c269a4d1a3d), while class 2 integron accession numbers were retrieved from the INTEGRALL database (last update: November 2017; http://integrall.bio.ua.pt). The corresponding nucleotide sequences were downloaded in FASTA format.

These sequences were then screened using a custom Python 3.10 script designed to search for DNA motifs corresponding to various fragments of the 3’ end of the *attI1* site.

Detected matches were manually verified and curated using SnapGene Viewer (https://www.snapgene.com/), allowing visual inspection of the gene cassette context and confirmation of unusual Δ*attI*-type elements. The complete script is available at https://github.com/xdrixnovsky/Trade-off-integrons.

To identify tandem gene cassettes in SCIs, we analyzed all 143 fully assembled RefSeq *Vibrio cholerae* genomes available in GenBank (April 2025). Integron’s locations were predicted using IntegronFinder 2.0^59^ and annotated integrons were downloaded in GenBank format. We then developed a Python 3.10 script to detect SCIs containing more than 1 identical *Vibrio cholerae* repeats (VCR) in their variable region. All outputs were manually verified using SnapGene Viewer. The complete script is available in https://github.com/xdrixnovsky/Trade-off-integrons.

Bar plots were created using R v4.5.1 (https://www.r-project.org/) and illustrations were made in Inkscape v1.3 (https://inkscape.org/).

## Supplementary Information

Below is the link to the electronic supplementary material.

Supplementary Material 1

## Data Availability

The datasets generated and/or analyzed during the current study are available in the National Center for Biotechnology Information, https://www.ncbi.nlm.nih.gov/ under the accession numbers: PX206332, PX206333, PX206334, PX257573, PX257573, PX206335 and PV877221.
