# Supplementary material for: Trade-off between canonical and unusual recombination sites promotes diversity and stability of gene cassette arrays of mobile integrons
Source: Sci Rep. 2026 Jan 24;16:6133. doi: 10.1038/s41598-026-36353-0 (PMC12901181; doi:10.1038/s41598-026-36353-0)
Supplement: Supplementary file 1 — Supplementary Material 1 [file 41598_2026_36353_MOESM1_ESM.pdf]

Supplementary information
Scientific Reports

"Trade-off between canonical and unusual recombination sites contributes to promote diversity and stability of gene cassette arrays of mobile integrons"

Adrián Gonzales Machuca, María Carolina Molina, Verónica Elizabeth Álvarez, Eduardo José Carpio Díaz, María Paula Quiroga* & Daniela Centrón*

Laboratorio de Investigaciones en Mecanismos de Resistencia a Antibióticos. Instituto de Investigaciones en Microbiología y Parasitología Médica (IMPaM). Facultad de Medicina, Universidad de Buenos Aires (UBA) - Consejo Nacional de Investigaciones Científicas y Técnicas (CONICET), Buenos Aires, Argentina.

***Corresponding authors.**

Mailing addresses: dcentron@gmail.com (Centrón, D.), quirogamp@gmail.com (Quiroga, M.P.)

Supplementary Table S1. Variable regions of class 1 and 2 integrons containing unusual Δ*attI*-type gene cassettes identified in Dr. Jové's and INTEGRALL databases.

Class 1 integrons gene cassette array	In Nº	Accession number
bla$_{OXA-101}$-ΔattI1$_{-11+6}$-aacA4'	In14	AM412777
aadA16-ΔattI1$_{-11+6}$-aacA4'-3-gcu130-catB3	In39	AY740681
bla$_{GES-1}$-ΔattI1$_{-11+6}$-aacA4'-dfrA15b-cmlA4-aadA2	In52	AF156486
qacH4-aadB-aacA1b-gcuG-bla$_{VEB-1}$-aadB-arr2-cmlA1f-bla$_{OXA-10}$-ΔattI1$_{-11+6}$-aadA1	In53	AF205943
bla$_{OXA-10}$-ΔattI1$_{-11+6}$-aacA4-bla$_{VIM-4}$	In108	AY509609
aadB-catB5-bla$_{OXA-10}$-ΔattI1$_{-11+6}$-aadA1-ΔattI2$_{-11+6}$-dfrA1b-ΔattI2$_{-10+6}$-aacA4'-8	In251	HQ386834
aadB-catB5-bla$_{OXA-10}$-ΔattI1$_{-11+6}$-aadA1	In252	HQ386837
aadB-bla$_{OXA-10}$-ΔattI1$_{-11+6}$-aadA1	In296	AY536742
bla$_{OXA-10}$-ΔattI1$_{-11+6}$-aadA1e-cmlA1g	In331	AY115475
catB3-bla$_{OXA-10}$-ΔattI1$_{-11+6}$-aadA15	In353	DQ393783
cmlA1g-bla$_{OXA-10}$-ΔattI1$_{-11+6}$-aadA1e	In355	EF034153
dfrA14d-aadA6-ΔattI1$_{-11+6}$	In387	FM877480
aadB-bla$_{VIM-2}$-aadA6-10-ΔattI1$_{-11+6}$	In461	AM087405
arr2-cmlA1g-bla$_{OXA-10}$-ΔattI1$_{-11+6}$-aadA1e	In467	EU886979
arr3-aacA4'-bla$_{OXA-10}$-ΔattI1$_{-11+6}$-aadA1e	In468	FM877487
bla$_{OXA-10}$-aadB-aadA10-ΔattI1$_{-11+6}$	In483	U37105
bla$_{OXA-74}$-ΔattI1$_{-11+6}$-aacA4cr2-cmlA1g	In484	EU161636
bla$_{VIM-2}$-bla$_{OXA-10}$-ΔattI1$_{-11+6}$-aacA4'-8	In493	AM392427
bla$_{VIM-6}$-bla$_{OXA-10}$-ΔattI1$_{-11+6}$-aacA4	In496	FM994936
gcu14-bla$_{GES-1}$-ΔattI1$_{-11+6}$-aacA4	In536	DQ236170
aadA6-ΔattI1$_{-11+6}$-aacA4'-17-smr2Δ-bla$_{OXA-10}$Δ	In570	AF453998
bla$_{VEB-1}$-aadB-arr2-cmlA1-bla$_{OXA-10}$-ΔattI1$_{-11+6}$-aadA1	In622	CT025832
bla$_{VIM-6}$-bla$_{OXA-10}$-ΔattI1$_{-11+6}$-aacA4-qacK-aadA1e	In632	EF522838
dfrA14b-arr2-cmlA1g-bla$_{OXA-10}$-ΔattI1$_{-11+6}$-aadA1e	In633	JN193567
aacA42-bla$_{GES-1}$-ΔattI1$_{-8+6}$-bla$_{GES-5}$-aacA4'-bla$_{OXA-2}$-qacH4-aadA1b	In647	GQ337064
arr3-qnrVC1c-bla$_{OXA-10}$-ΔattI1$_{-11+6}$-aadA1bc	In704	HM015626
qnrVC1-bla$_{OXA-10}$-ΔattI1$_{-11+6}$-aadA1bc	In703	HM015625
aacA42-bla$_{GES-19}$-ΔattI1$_{-11+6}$-bla$_{GES-20}$-aacA4'-bla$_{OXA-2}$-qacH4-aadA1	In724	JN596280
aadB-gcuE2-gcu8-cmlA1g-bla$_{OXA-10}$-ΔattI1$_{-11+6}$-aadA1e	In752	JN687470
bla$_{OXA-10}$-ΔattI1$_{-11+6}$-aacA4-bla$_{VIM-2}$-arr2-dfrB2b	In778	JQ629929
qnrVC4-qacH4-aacA4-17-cmlA1g-bla$_{OXA-10}$-ΔattI1$_{-11+6}$- aadA1e-dfrA14b	In805	KU886277
bla$_{IMP-1}$-aadA24-ΔattI1$_{-11+6}$-aacA4	In828	AB901036
qacH4-bla$_{OXA-10}$-ΔattI1$_{-11+6}$-aadA1	In834	JX141473
dfrA14b-arr2-cmlA1Δ2-bla$_{OXA-10}$Δ2-ΔattI1$_{-11+6}$-aadA1a	In869	AJ971342
bla$_{IMP-1}$-dfrB9-aacA4'-smr2-bla$_{OXA-10}$-ΔattI1$_{-11+6}$-aadA1-cmlA1g	In878	KC675185
aadB-bla$_{OXA-10}$-ΔattI1$_{-11+6}$-aadA1cc	In909	HG428757
aacA4-aadA1-ΔattI1$_{-11+6}$-bla$_{OXA-9}$	In907	HG428757
bla$_{OXA-10}$-ΔattI1$_{-11+6}$-aadA1cg	In941	KF525298
dfrA14b-arr2-bla$_{OXA-10}$-ΔattI1$_{-11+6}$-aadA1e	In1080	KM111261
dfrB4-catB3-bla$_{OXA-10}$-ΔattI1$_{-11+6}$-aadA1a	In1134	KM595289
aacA4'-bla$_{OXA-142}$-aadA6-ΔattI1$_{-11+6}$	In1161	EU358785
dfrA14-arr2-cmlA7-bla$_{OXA-10}$-ΔattI1$_{-11+6}$-aadA1	In1166	LC055503

arr3-cmlA1g-bla$_{OXA-10}$-ΔattI1$_{-11+6}$-aadA1e	In1169	LN831185
bla$_{VIM-2}$-ΔattI1$_{-10+6}$-aacA4-bla$_{OXA-10}$-aadA1a	In1198	KR259332
arr3-qnrVC1-bla$_{OXA-10}$-ΔattI1$_{-11+6}$-aadA1e	In1214	KR827393
qnrVC4-qacF-aacA4'-17-cmlA1g-bla$_{OXA-10}$-ΔattI1$_{-11+6}$-aadA1e-dfrA14b	In1222	KU160531
bla$_{OXA-10}$-ΔattI1$_{-11+6}$-aacA4-bla$_{IMP-55}$-cmlA1g	In1243	KT935306
aacA7-bla$_{GES-1}$-aadB21-ΔattI1$_{-12+6}$	In1267	KU544301
aadB-bla$_{OXA-10}$-ΔattI1$_{-11+6}$-aacA4	In1295	KU839731
qnrVC4-aacA4-17-cmlA1g-bla$_{OXA-10}$- ΔattI1$_{-11+6}$-aadA1e-dfrA14b	In1300	CP022170
bla$_{VEB-2}$-aadB-arr2-cmlA1g-bla$_{OXA-10}$-ΔattI1$_{-11+6}$-aadA1e	In1307	KU356480
bla$_{OXA-10}$-ΔattI1$_{-11+6}$-aacA4-bla$_{IMP-1}$-qacG10	In1313	LC169568
bla$_{IMP-14}$-bla$_{OXA-10}$-ΔattI1$_{-11+6}$-aacA4	In1314	LC169569
aadB-aadA17b-bla$_{OXA-10}$-ΔattI1$_{-11+6}$-aadA1de	In1341	KU728263
arr3-cmlA1g-bla$_{OXA-10}$-ΔattI1$_{-11+6}$-aadA1e-dfrA14b	In1352	CP038456
bla$_{OXA-10}$-ΔattI1$_{-11+6}$-aacA4-22	In1370	LC224309
aacA4'-bla$_{OXA-10}$-ΔattI1$_{-11+6}$-aadA11c-qacM	In1397	LT882698
aacA4'-gcuE29-bla$_{OXA-10}$-ΔattI1$_{-11+6}$-aadA1a	In1415	KY913898
dfrA14-arr2-cmlA5-bla$_{OXA-10}$-ΔattI1$_{-11+6}$-aadA1	In1419	CP021853
arr2D-cmlA1g-bla$_{OXA-10}$-ΔattI1$_{-11+6}$-aadA1e	In1422	CP021958
aacA4'-8-bla$_{VIM-1}$-bla$_{OXA-10}$-ΔattI1$_{-11+6}$-aacA4-14	In1434	MF687203
aacA4'-8-bla$_{VIM-1}$-Δbla$_{OXA-10}$-IS26-Δbla$_{OXA-10}$-ΔattI1$_{-11+6}$-aacA4-14	In1435	MF687204
aadB-bla$_{OXA-10}$-ΔattI1$_{-11+6}$-aadA1	In1437	KY884677
aacA4-5-qacG-bla$_{IMP-1}$-aadA24-ΔattI1$_{-11+6}$-aacA4-5	In1477	AP018455
bla$_{IMP-48}$-aadB-bla$_{OXA-10}$-ΔattI1$_{-11+6}$-aacA4	In1491	KY574887
aadB-bla$_{IMP-1}$9-aadA6-ΔattI1$_{-11+6}$	In1518	MH071810
arr2-cmlA1g-bla$_{OXA-10}$-ΔattI1$_{-11+6}$-aadA1	In1526	MF113045
qnrVC4-qacF-bla$_{OXA-10}$-ΔattI1$_{-11+6}$-aadA1e-dfrA14b	In1567	CP032238
bla$_{OXA-10}$-ΔattI1$_{-11+6}$-aacA4-bla$_{IMP-1}$	In1593	CP031449
aacA7-aadB-aacA7-dfrB2-bla$_{OXA-10}$-ΔattI1$_{-11+6}$-aadA1e	In1599	CP033439
bla$_{GES-1}$-ΔattI1$_{-11+6}$-aacA4-cmlA4-aadA2	In1600	NZ_QORA01000012
bla$_{OXA-10}$-ΔattI1$_{-11+6}$-aacA4-bla$_{VIM-2}$-arr2-dfrB2-cmlA1g-bla$_{OXA-10}$-ΔattI1$_{-11+6}$-aadA1e	In1617	LR134330
gcu190-gcu191-bla$_{VIM-2}$-aadB-aadA6-ΔattI1$_{-11+6}$	In1640	MW595314
bla$_{VIM-2}$-aadA6-ΔattI1$_{-11+6}$	In1650	MW595324
gcu193-bla$_{OXA-10}$-ΔattI1$_{-11+6}$-aacA4'-bla$_{VIM-2}$-aadA6-ΔattI1$_{-11+6}$	In1656	MW595330
gcu193-2-bla$_{OXA-10}$-ΔattI1$_{-11+6}$-aacA4'-bla$_{VIM-2}$-aadA6-ΔattI1$_{-11+6}$	In1657	MW595331
arr2-gcu196-cmlA1g-bla$_{OXA-10}$-ΔattI1$_{-11+6}$-aadA1e	In1664	CP035739
aacA4-14-bla$_{IMP-1}$-aadA6-ΔattI1$_{-11+6}$	In1668	MW595334
bla$_{OXA-10}$-ΔattI1$_{-11+6}$-aadA1	In1672	MG878868
bla$_{IMP-4}$-qacG2-aacA4'-catB3-bla$_{OXA-10}$-ΔattI1$_{-11+6}$-aadA1a-bla$_{VEB-3}$	In1766	CP042480
aacA27-bla$_{OXA-10}$-ΔattI1$_{-11+6}$-qacE	In1769	MN961670
bla$_{VIM-2}$-aadA2-ΔattI1$_{-11+6}$-bla$_{OXA-10}$-ΔattI1$_{-11+6}$-aacA4	In1770	CP045553
bla$_{OXA-10}$-ΔattI1$_{-11+6}$-aadA1e	In1815	CP096946
aadB-bla$_{OXA-10}$-ΔattI1$_{-11+6}$-aadA1e-qnrVC1-aacA4'	In1818	CP096932
bla$_{OXA-10}$-ΔattI1$_{-11+6}$-aadA1e-qnrVC1-aacA4'-aadB	In1836	CP096937
gcu210-aacA4'-bla$_{OXA-17}$-aadA6-ΔattI1$_{-11+6}$	In1852	OR178458
aadA6-10-ΔattI1$_{-11+6}$-aacA4'-smr2-aadA7e-bla$_{VIM-2}$	In1894	MW595335
bla$_{OXA-10}$-ΔattI1$_{-11+6}$-aadA1e-qnrVC1-aacA4'	In2044	CP096929
dfrA14b-arr2-bla$_{OXA-10}$-ΔattI1$_{-11+6}$-aadA1a-ΔattI2$_{-11+6}$-dfrA1b-ΔattI2$_{-10+6}$-aacA4	In2123	MT813046
bla$_{OXA-10}$-ΔattI1$_{-11+6}$-aacA4	In2170	CP097249
bla$_{VIM-2}$-aacC11b-aadA6-ΔattI1$_{-11+6}$	In2242	OQ116831
bla$_{IMP-19}$-aacA4'-8-aadA1b-catB2-ΔattI1$_{-11+6}$	In2241	OQ116830
**Class 2 integrons gene cassette array**	**In N°**	**Accession number**
dfrA1-sat2-ybeA-ΔattI2$_{-11+6}$	In2-3	JX867127
dfrA1-sat2-aadB4-catB2-ΔattI2$_{-238+6}$-dfrA1-blaCARB-4-aadA1y	In2-5	FJ785525
sat2-aadB4-catB2-ΔattI2$_{-238+6}$-dfrA1-aadA1y-ybeA-ΔattI2$_{-11+6}$	In2-8	DQ176450
linF2-ΔattI2$_{-9+6}$-dfrA1-ΔattI2$_{-10+6}$-aadA1a	In2-16	HQ386831
dfrA1k-sat2-aadA1y-ybeA-ΔattI2$_{-11+6}$	In2-43	FJ591049
dfrA1k-sat2e-aadA1as-ybeA-ΔattI2$_{-11+6}$	In2-44	FJ591055
dfrA1Δ-sat2-aadA1cy-ybeA-ΔattI2$_{-11+6}$	In2-52	LN827615
aadA34-ybeA-ΔattI2$_{-11+6}$	In2-66	CP012140

Supplementary Table S2. Recombination frequencies mediated by IntI1. ND: Not determined. [1]Frequencies represent the average of 30 randomly selected colonies (technical replicates) from the induced cultures. The entire experiment was performed in three biological replicates (n=3), except pACA3 which used six biological replicates (n=6).

Plasmids	Gene cassette architecture	Excision frequency (%)[1]		Cut positions (5'/3' ends)
pAO1B$_{-11}$	*attI1-aadB–ΔattI1*$_{-11}$	4		G'TTAGGC/G'TTAGAT
pORFX	*attC*$_{aadA1}$*-ybeA-ΔattI2*$_{-11}$	28		G'TTAGAG/G'TTAGGC
paadB-*attI2*$_{-238}$	*attC*$_{\text{aadA1}}$*-aadB-ΔattI2*$_{-238}$	80		G'TTAGGC/G'TTATGA
		20		G'TTAGGC/G'TTAACC
pAO1B1	*attI1-aadB-attI*	100		G'TTAGGC/G'TTAGAT
pAO1Bc	*attI1-aadB-attC*$_{aadB}$	71		G'TTAGGC/G'TTAGAT
pAO1BΔC	*attI1-aadB-ΔattC*$_{aadB}$	0		(-)/(-)
pAcBc	*attC*$_{sat2}$*-aadB-attC*$_{aadB}$	49		G'TTAGGC/G'TTAGGC
pACA3	*attC*$_{aadB}$*-aadB-attC*$_{aadB}$	97		G'TTAGGC/G'TTAGGC
**Plasmids**	**Gene cassette architecture**	**Insertion frequency (%)[1]**		**Cut positions (5'/3' ends)**
		**5'**	**3'**	
pAO1B$_{-11}$	*attI1-aadB–ΔattI1*$_{-11}$	8	7	G'TTAGGC/G'TTAGAT
pORFX	*attC*$_{aadA1}$*-ybeA-ΔattI2*$_{-11}$	ND	ND	ND
paadB-*attI2*$_{-238}$	*attC*$_{aadA1}$*-aadB-ΔattI2*$_{-238}$	ND	ND	ND
pAO1B1	*attI1-aadB-attI1*	7	7	G'TTAGGC/G'TTAGAT
pAO1Bc	*attI1-aadB-attC*$_{aadB}$	4	9	G'TTAGGC/G'TTAGAT
pAO1BΔC	*attI1-aadB-ΔattC*$_{aadB}$	4	0	G'TTAGGC/ (-)
pAcBc	*attC*$_{sat2}$*-aadB-attC*$_{aadB}$	11	6	G'TTAGGC/G'TTAGGC
pACA3	*attC*$_{aadB}$*-aadB-attC*$_{aadB}$	18	13	G'TTAGGC/G'TTAGAT

Supplementary Table S3. Tandem gene cassettes arrays detected in the variable region of class 1 integrons and in sedentary chromosomal integrons. No tandem gene cassette arrays were identified in class 2 integrons.

Tandem gene cassettes arrays detected in class 1 integrons	GC repeated	Times repeated	In number	Accession number
bla$_{\text{VIM-1}}$*-aacA4'-aacA4'-bla*$_{\text{OXA-46}}$	*aacA4*	2	In80	AJ969237
gcu114-gcu114-aadA6h	*gcu114*	2	In539	GQ281704
dfrB4c-aacA4'-aacA4'-aadA1e	*aacA4'*	2	In602	HQ317921
aacC1-gcuP-gcuP-gcuQ-aadA1a	*gcuP*	2	In616	AY922990
aacC1-gcuP-gcuP-gcuQ-aadA1ay	*gcuP*	2	In617	AY577724
aacC1e-gcuP-gcuP-gcuQ-aadA1b	*gcuP*	2	In618	AF453999
dfrA12-gcuF-aadA2-aadA2-cmlA1-aadA1a-qacH2	*aadA2*	2	In649	EF113389
aadA7-aadA7	*aadA7*	2	In674	FJ980455
bla$_{\text{IMP-1}}$*-bla*$_{\text{IMP-1}}$*-gcu130-catB3-bla*$_{\text{IMP-1}}$	*bla*$_{\text{IMP-1}}$	2	In802	AB698823
bla$_{\text{IMP-1}}$*-aacA4'-3-aacA4'-3-catB6-bla*$_{\text{CARB-12}}$	*aacA4'-3*	2	In827	AB901035
aacC1-gcuP-gcuP-gcuP-gcuQ-aadA1a	*gcuP*	3	In906	CP003500
smr1-aacA4'-3-aacA4'-3	*aacA4'-3*	2	In912	KF556707
bla$_{\text{VIM-4}}$*-bla*$_{\text{VIM-4}}$	*bla*$_{\text{VIM-4}}$	2	In1173	KP975074
fosL-fosL-bla$_{\text{GES-5}}$	*fosL*	2	In1187	KT070138
aacA8-bla$_{\text{OXA-2}}$*-aacA7-aacA7-aadA6-gcuDD*	*aacA7*	2	In1380	CP020603
dfrB5-arr2-dfrB5-dfrB5-bla$_{\text{OXA-129}}$	*dfrB5*	2	In1594	CP031449
qacG-qacG	*qacG*	2	In1908	MN418915
gcu228-gcu228	*gcu228*	2	In1944	MN418959
aacA7-blaGES-4-bla$_{\text{GES-24}}$*-bla*$_{\text{GES-24}}$*-bla*$_{\text{GES-24}}$-AP021918-*catB6D*-AP021918	*bla*$_{\text{GES-24}}$	2	In1972	AP021918
aacA7-blaGES-4-bla$_{\text{GES-24}}$*-bla*$_{\text{GES-24}}$*-bla*$_{\text{GES-1D}}$-AP021921-*catB6D*-AP021921	*bla*$_{\text{GES-24}}$	2	In1973	AP021921
bla$_{\text{GES-24}}$*-bla*$_{\text{GES-24}}$*-bla*$_{\text{GES-24}}$*-bla*$_{\text{GES-24}}$*-aacA4'-3*	*bla*$_{\text{GES-24}}$	4	In1976	AP024404
fosE-aacA4'-3-aacA4'-3-bla$_{\text{IMP-1}}$*-qacG*	*aacA4'-3*	2	In2025	AP024354
aacA4'-AP024402-*bla*$_{\text{IMP-1}}$*-aacA4'*-AP024402-*aacA4'*-AP024402-*bla*$_{\text{OXA-1}}$*-aacA4'-3*	*aacA4'*-AP024402	2	In2026	AP024402
bla$_{\text{GES-24}}$*-bla*$_{\text{GES-24}}$- IS*Spu7**-bla*$_{\text{GES-24}}$*-gcu238-bla*$_{\text{OXA-921}}$	*bla*$_{\text{GES-24}}$	2	In2067	LC621169
aacA4'-aacA4'-aacA4'-aacC1-MZ215787*, qacH, bla*$_{\text{OXA-4}}$	*aacA4'*	3	In2107	MZ215786
fosE-aacA4'-3-aacA4'-3-bla$_{\text{IMP-1}}$	*aacA4'-3*	2	In2111	AP024942
bla$_{\text{VIM-2}}$*-bla*$_{\text{VIM-2}}$	*bla*$_{\text{VIM-2}}$	2	In2133	OL880464
bla$_{\text{VIM-2}}$*-bla*$_{\text{VIM-2}}$*-gcuQ*	*bla*$_{\text{VIM-2}}$	2	In2136	OL880467
**Tandem gene cassettes arrays detected in sedentary chromosomal integrons**	**GC repeated**	**Times repeated**	**In number**	**Accession Number**
dfrA31-dfrA31-dfrA31'	*dfrA31*	3	-	NZ_CP063421

- Gene cassette screening in class 1 integrons was performed using Dr. Jové's database (April 2025 release), while superintegrons analysis utilized all 143 *Vibrio cholerae* closed genomes available in GenBank (as of 28 April 2025).
- Gene cassettes marked with accession numbers may contain sequence modifications. For complete annotation details, refer to Dr. Jové's tables (https://annuel2.framapad.org/p/r.461a5f838700ae34c5d12c269a4d1a3d).
- (*) Unreported element in the reference dataset.

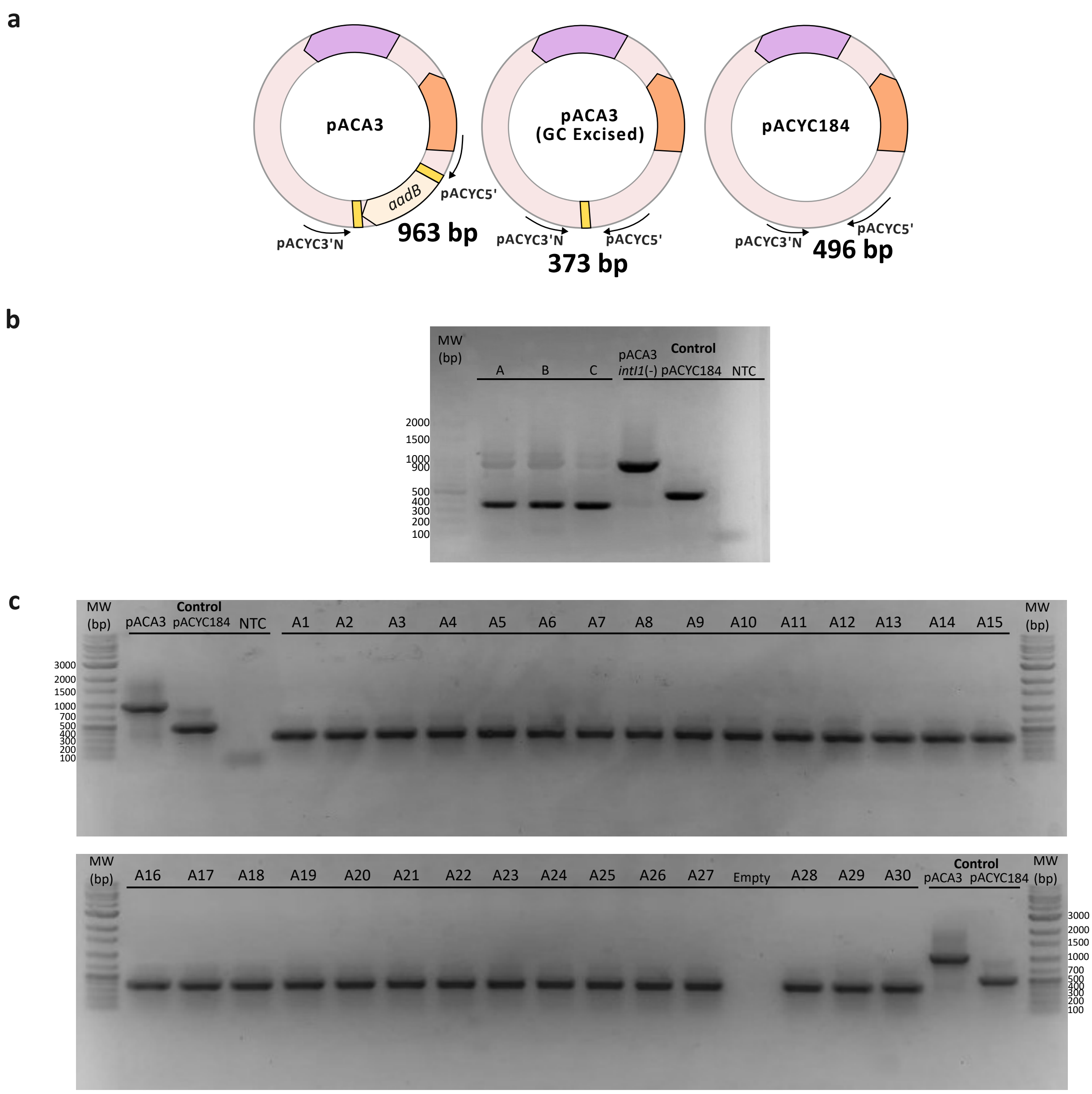

Supplementary Figure S1. IntI1-mediated excision of the *attC*$_{aadB}$-*aadB*-*attC*$_{aadB}$ gene cassette from plasmid pACA3. (a) Schematic representation of the plasmids and expected PCR products. The diagram shows the synthetic plasmid pACA3 containing the *attC*$_{aadB}$-*aadB*-*attC*$_{aadB}$ gene cassette array. If no excision occurs, the template size is 963 bp (pACA3). After successful site-specific excision mediated by IntI1, the resulting plasmid (GC Excised) should yield a PCR product of 373 bp. The control vector, pACYC184 (the backbone used for subcloning), is shown with an expected product size of 496 bp. The positions of the primers (pACYC3'N and pACYC184-5') used for the excision PCR analysis are indicated by black arrows. (**b) Excision assay verification by PCR.** The gel shows the PCR products obtained using plasmid DNA extracted from three biological replicates (A, B, and C) of the *in vivo* excision assay for *E. coli* co-transformed with pACA3 and the IntI1-expressing plasmid pLQ369. The predominant 373 bp band indicates a high frequency of IntI1-mediated excision. Controls include pACA3-intI1(-) (a recombination assay carried out only with pACA3, without pLQ369 co-transformation), plasmid pACYC184 DNA (496 bp control band), and a No Template Control (NTC). Molecular weight markers (MW) are shown on the left. **(c) Colony PCR analysis of transformed *E. coli* TOP10 (*recA1*) cells.** Colony PCR was performed on 30 randomly selected colonies (A1 to A30) transformed with the plasmid DNA extracted from replicate A shown in panel (b). The prevalence of the smaller 373 bp band (excised gene cassette) over the 963 bp band (non-excised gene cassette) confirms the high excision frequency. Controls include pACA3 plasmid DNA, pACYC184 plasmid DNA, and a NTC.

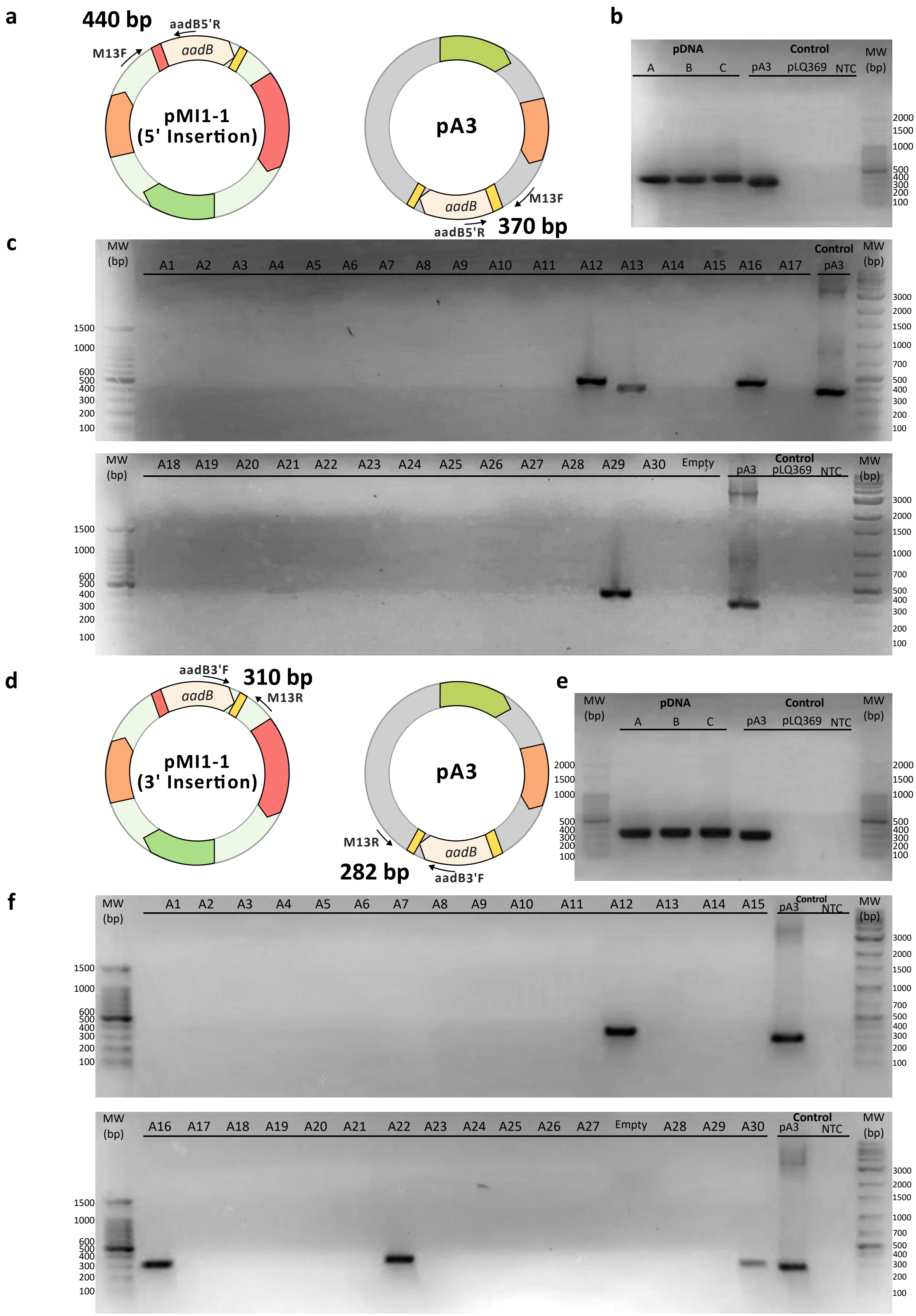

Supplementary Figure S2: IntI1-mediated insertion of *attC$_{aadB}$-aadB-attC$_{aadB}$* gene cassette from plasmid pACA3 into the pMI1-1 *attI1* site. Control plasmid pA3 (pMG-Kan-Macrogen, pUC derivative) was used as a template for primer controls. **(a-c) 5' Insertion Analysis**. Panel **(a)** shows the schematic diagram of the expected pMI1-1 insertion product (440 bp) and the pA3 control (370 bp) amplified using primers M13F and aadB5'R (black arrows). Panel **(b)** shows the PCR of plasmid DNA from biological replicates (A, B, C) confirming the successful 5' insertion product. pA3 was used as positive control, while pLQ369 was used as a negative control for insertion, as it lacks the *attI1* site. No Template Control (NTC) and molecular weight markers (MW) are also shown on each gel. Panel **(c)** shows the Colony PCR analysis of 30 randomly selected colonies. The 440 bp band indicates insertion, and the 370 bp band indicates the pA3 control product. **(d-f) 3' Insertion Analysis**. Panel **(d)** shows the schematic diagram of the pMI1-1 insertion product (310 bp) and the pA3 control (282 bp) amplified using primers aadB3'F and M13R. Panel **(e)** shows the PCR of plasmid DNA from biological replicates (A, B, C) confirming the successful 3' insertion product, with pA3 and pLQ369 serving as controls. Panel **(f)** shows the Colony PCR analysis of 30 randomly selected colonies. The 310 bp band indicates insertion, and the 282 bp band indicates the pA3 control product.

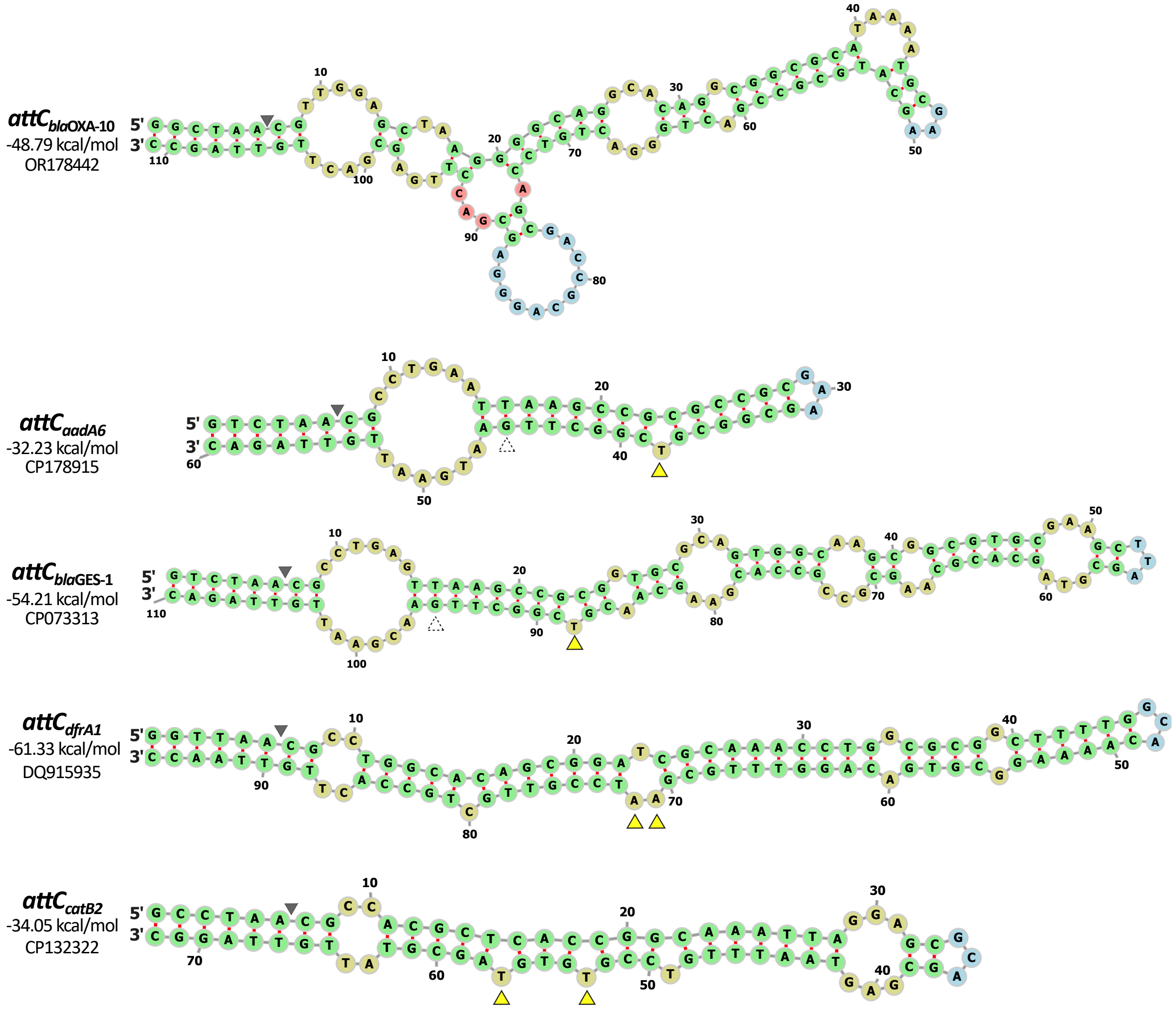

Supplementary Figure S3. Predicted secondary structures of the *attC* sites associated with prevalent unusual Δ*attI*-type gene cassettes. The minimum free energy secondary structures of the bottom strands were generated using RNAfold. The analysis includes *attC* sites from $bla_{\text{OXA-10}}$, *aadA6*, $bla_{\text{GES-1}}$, *dfrA1*, and *catB2* gene cassettes, with their respective thermodynamic stability values (ΔG, kcal/mol) and GenBank accession numbers indicated on the left. Grey arrowheads mark the recombination crossover point within the R-box. Yellow arrowheads indicate the position of extrahelical bases (EHBs) in the structures where they are preserved while dotted arrowheads indicate possible EHBs. Notably, the $attC_{\textbf{bla}\text{OXA-10}}$ and $attC_{aadA6}$ sites exhibit structural deviations, such as the loss of canonical EHBs or aberrant stem-loop folding; these structural defects are proposed to impede the formation of the functional single-stranded synaptic complex, thereby biasing the system toward the double-stranded recombination pathway that might be involved in the Δ*attI* sites generation.
